# Survivin recombinant overlapping peptide (ROP) vaccine in advanced solid tumours: a first-in-human, multicentre, open-label, phase 1a dose-escalation study

**DOI:** 10.1016/j.eclinm.2025.103717

**Published:** 2025-12-27

**Authors:** Wynne Wijaya, Thomas Morris, Martin D. Forster, Michael Flynn, Mark Tuthill, Fiona Thistlethwaite, Anja Williams, William Finch, Wenshu Lu, Shisong Jiang

**Affiliations:** a Department of Oncology, University of Oxford, Oxford, Oxfordshire, UK; b Oxford Vacmedix, Oxford, Oxfordshire, UK; c University College London Cancer Institute/University College London Hospitals NHS Trust, London, UK; d Oxford University Hospitals NHS Trust, Oxford, Oxfordshire, UK; e The Christie NHS Foundation Trust, Manchester, UK; f Sarah Cannon Research Institute, London, UK

**Keywords:** Immunotherapy, Cancer vaccine, Peptide-based vaccine, Survivin, BIRC5, Inhibitor of apoptosis protein, Recombinant overlapping peptides

## Abstract

**Background:**

Survivin, an inhibitor of apoptosis protein (IAP), is highly expressed in various cancers but has weak immunogenicity as a self-derived tumour-associated antigen (TAA). OVM-200, a survivin recombinant overlapping peptide (ROP) vaccine, consists of overlapping peptides linked by the target sequence (LRMK) for cathepsin S, preserving T-cell and most antibody epitopes. OVM-200 elicits both cellular and humoral immune responses against survivin-expressing cancer cells. This phase 1a, multicentre, open-label trial (OVM-200-100) evaluates OVM-200 as a therapeutic vaccine in patients with non-small cell lung, ovarian, and prostate cancer. This Phase 1 trial is also the first time the ROP technology platform has been used in human trials.

**Methods:**

Twelve eligible patients received three subcutaneous OVM-200 doses at 2-week intervals using a 3 + 3 dose-escalation design. Four dose levels (250, 500, 1000, and 2000 μg) were tested to determine the optimal dose for phase 1b. The primary endpoint was safety and tolerability, while secondary endpoints included immunogenicity (antibody and T-cell response) and tumour response (RECIST criteria). This trial was registered with ClinicalTrials.gov (NCT05104515) and EudraCT (2021-001545-12) and took place from 28/09/2021 to 04/05/2023.

**Findings:**

OVM-200 was well tolerated, with no serious adverse drug reactions (ADRs) or dose-limiting toxicities (DLTs). All adverse events were Grade 1 injection site reactions (ISRs). The 2000 μg dose group achieved the highest median anti-survivin IgG titre (1:327,680) at the end of the study (EOS) and a median ELISpot T cell response of 1282 SFU per million cells on day 22. Disease stabilisation (SD) was observed in 6 of 12 patients (50%), including all 3 patients (100%) in the 2000 μg group, some of which were stabilisations of limited duration. Based on these findings, the 2000 μg dose was selected for further evaluation in phase 1b.

**Interpretation:**

OVM-200 is well tolerated and induces a robust humoral response, with a considerable cellular response and preliminary evidence of disease stabilisation. Phase 1b is ongoing to further evaluate its safety and efficacy at the selected dose.

**Funding:**

Oxford Vacmedix UK Ltd.

**Research in context**

**Evidence before this study**

We searched PubMed, Embase, ClinicalTrials.gov, and references from relevant reviews for studies published between January 1, 2000, and May 1, 2024, using combinations of the following terms: “survivin”, “peptide vaccine”, “cancer vaccine”, “recombinant overlapping peptide”, “immunotherapy”, and “clinical trial”. We included preclinical and clinical studies, regardless of language, that investigated survivin-based peptide vaccines in human cancers. Several peptide vaccines targeting survivin, including SurVaxM, demonstrated safety and immunogenicity in early-phase trials for glioblastoma and neuroendocrine tumors, although their efficacy was restricted to specific HLA haplotypes (HLA-A∗02, A∗03, or A∗24). We found no prior clinical trials using recombinant overlapping peptide (ROP) technology in humans. Previous studies identified limited T-cell response persistence and modest clinical efficacy as challenges for peptide-based vaccines.

**Added value of this study**

This study is the first-in-human evaluation of OVM-200, a survivin recombinant overlapping peptide vaccine targeting survivin. Unlike earlier survivin vaccines, OVM-200 is designed to stimulate a broader immune response across diverse HLA haplotypes, potentially expanding its applicability. Our findings contribute novel clinical evidence on the safety, tolerability, and immunogenic potential of ROP-based vaccines and provide preliminary data supporting their further development.

**Implications of all the available evidence**

Taken together with previous evidence on survivin vaccines, this study supports the continued development of HLA-unrestricted peptide-based cancer vaccines. The safety and immunogenicity profile of OVM-200 suggests its potential as a component in multi-modal immunotherapy strategies, including combination with immune checkpoint inhibitors, immunostimulants, or cytokine therapies. The ongoing phase 1b trial may clarify its clinical efficacy and provide guidance on optimal booster regimens and combination strategies for subsequent phases of clinical trials.

## Introduction

Survivin is an inhibitor of apoptosis protein (IAP) that is overexpressed in approximately 90 percent of human cancers^1^, ^2^, ^3^, ^4^ and is associated with tumour progression and poor prognosis,^3^^,^^5^, ^6^, ^7^, ^8^, ^9^, ^10^, ^11^ making survivin an attractive target for cancer therapy.^3^^,^^12^ However, in its native form, survivin is weakly immunogenic. Survivin-based vaccines have shown promising results in inducing cytotoxic T lymphocyte (CTL) response against survivin-expressing cancer cells both *in vitro* and *in vivo*.^13^ A survivin peptide-based vaccine, SurVaxM, has also demonstrated safety, tolerability, and immunogenic effects in human patients with malignant gliomas and metastatic neuroendocrine tumours (NETs) in numerous clinical trials.^14^, ^15^, ^16^, ^17^ However, the immunogenicity of this vaccine may be limited and only to certain HLA haplotypes, such as HLA-A∗02, HLA-A∗03, and HLA-A∗24 due to the nature of its design.^14^ To overcome these limitations, we developed a recombinant overlapping peptide (ROP) vaccine, OVM-200, in which the full-length survivin protein is expressed as a series of overlapping peptides linked by a cathepsin S cleavage motif (LRMK). This design facilitated efficient intracellular processing by antigen-presenting cells and enhances presentation of multiple survivin epitopes on both MHC Class I and II molecules, thereby broadening CD4^+^ and CD8+ T cell recognition independent of HLA type. Moreover, ROP enables efficient industrial manufacture, since it can be produced in *E. coli* and developed in multiple formats, including DNA, mRNA, and vector-based platforms.

Our previous studies have demonstrated the immunological advantages of the ROP approach. Cai et al. showed that ROP-pulsed dendritic cells stimulated stronger CD8^+^ T cell responses from human PBMCs compared with wild-type survivin.^18^ Zhang et al. performed bioinformatic analyses confirming that survivin is widely expressed across human cancers. In vivo experiments also demonstrated that survivin ROP vaccination elicited robust CD4^+^ and CD8^+^ T cell immune responses and prolonged survival compared to the native survivin protein in a survivin-expressing melanoma mouse model.^19^ These findings provide strong preclinical evidence supporting the clinical evaluation of survivin ROP vaccination.

This first-in-human phase 1a, multicentre, open-label, non-randomized trial (OVM-200-100) represents the first use of the ROP technology platform in human trials. It primarily aims to evaluate the safety and tolerability of OVM-200 in patients with non-small cell lung cancer (NSCLC), ovarian cancer, and prostate cancer. As secondary endpoints, this study also attempts to determine the immunogenicity, tumour response, and the recommended dose of OVM-200 for further developments.

## Methods

### Study design

This study comprises a first-in-human (FIH) multiple-dose, sequential-cohort 3 + 3 design to establish a dose of OVM-200 that is safe and tolerable, and that elicits an immune response in humans. This dose will be taken forward into the second part of the study (phase 1b) of the study. Phase 1b will further assess the safety and tolerability of the selected dose and investigate the immune and anti-tumour response in 3 expansion cohorts of additional patients with NSCLC, ovarian cancer, and prostate cancer.

### Participants and eligibility

Eligibility criteria are: (1) aged between 18 and 75 years; (2) histologically confirmed metastatic or locally advanced inoperable NSCLC, ovarian cancer, or prostate cancer that have already received at least 1 line of approved cancer therapy and either: exhausted current recognized treatment options; or are stable in a planned treatment-free interval following completion of a set course of treatment; or in the case of prostate cancer, are currently stable on an antihormonal treatment; (3) patients are not receiving active cancer treatment other than supportive therapies or androgen deprivation therapies for prostate cancer, which may be continued, and, in the opinion of the investigator, are not anticipated to require further approved cancer treatment options until the Week 8 assessment (up to 9 weeks) after the first dose of OVM-200 per standard of care; (4) at least 1 measurable lesion that can be accurately assessed at baseline by computed tomography (CT)/magnetic resonance imaging (MRI) and is suitable for repeated assessment (NSCLC only); (5) predicted life expectancy of 3 months or longer; (6) adequate bone marrow, renal, and hepatic function.

**Fig. 1:**
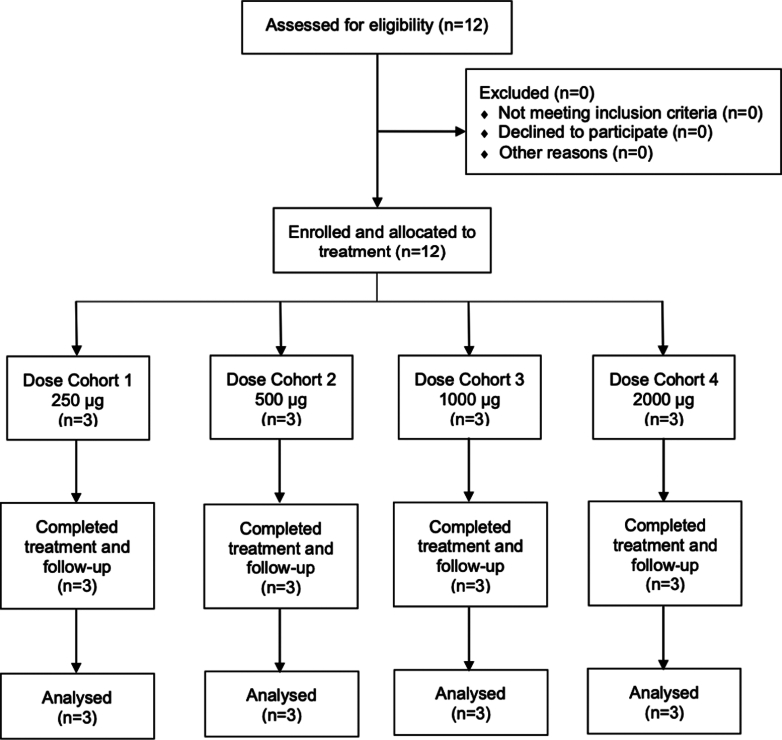
CONSORT flow diagram.

Patients were excluded if they have one of these criteria: (1) known history or evidence of significant immunodeficiency due to underlying illness, including patients with a condition requiring systemic treatment with either corticosteroids (>10 mg daily prednisolone equivalent) or other immunosuppressive medications within 14 days of the first dose of study drug; (2) a history of active, known, or suspected autoimmune diseases or a syndrome that requires systemic or immunosuppressive agents; (3) prior anticancer vaccine therapy, anti-PD-1, anti-PD-L1, anti-PD-L2, anti-CD137, anti-CTLA-4 antibody, or any other antibody or drug specifically targeting T-cell co-stimulation or immune checkpoint pathways in the 28 days before the first dose of study drug; (4) administration of an investigational drug in the 28 days or 6 half-lives (whichever is longer) before the first dose of vaccination; (5) major surgery or treatment with any chemotherapy or radiation therapy for cancer in the 28 days before the first dose of study drug; (6) active infection requiring antibiotics or physician monitoring, or recurrent fevers (>38 °C) associated with a clinical diagnosis of active infection; (7) active viral diseases; (8) receipt of any vaccine within 28 days before the first dose of study drug; (9) other prior malignancies within the previous 3 years; (10) symptomatic brain metastases or any leptomeningeal metastasis; (11) any serious or uncontrolled medical disorders; and (12) history of allergic reaction or hypersensitivity to any component of the OVM-200 vaccine or adjuvant. This study was registered in ClinicalTrials.gov (NCT05104515) and EudraCT (2021-001545-12). Study and recruitment took place from 28/09/2021 to 04/05/2023. Participant flow, including screening, enrolment, treatment completion, and follow-up, is shown in the CONSORT diagram (Fig. 1).

### Vaccine manufacture

OVM-200 is produced by Eurofins CDMO (Ghent, Belgium) under Good Manufacturing Practice (GMP) conditions using recombinant overlapping peptide synthesis. The peptides are designed to span the full length of survivin, ensuring broad MHC presentation. The final formulation includes an adjuvant, Montanide (Seppic, France), to enhance immune activation. Quality control is performed via HPLC and mass spectrometry to confirm peptide purity and stability. OVM-200 has been shown to be stable for four years under standard storage conditions.

### Vaccination

OVM-200 vaccine preparations were in the form of 2 mg/mL solution emulsified with the adjuvant at 1:1 ratio and were administered as a subcutaneous injection. The proposed doses for phase 1a were: 250 μg, 500 μg, 1000, and 2000 μg. The planned doses were adjusted based on the scientific review committee (SRC) recommendations. The dose for phase 1b was selected following review of the phase 1a data and was not to exceed the dose safely administered in phase 1a.

**Fig. 2:**
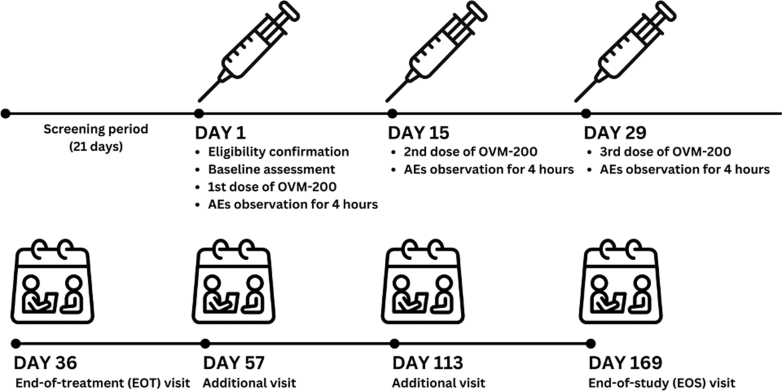
Timeline of study.

After a screening period of up to 21 days (Fig. 2), eligibility was confirmed, pre-vaccination baseline assessments completed, and eligible subjects received the first dose of OVM-200. Subjects received 3 doses of OVM-200 at 2-week intervals. Subjects were observed for at least 4 h after each dose and reviewed weekly through to the end-of-treatment (EOT) visit on Week 5 (Day 36). There were follow-up visits on Weeks 8 (Day 57) and 16 (Day 113), and an end-of-study (EOS) visit on Week 24 (Day 169). Participants remained on study for up to 6 months incorporating a total of 10 hospital visits. This study was conducted at four sites in the United Kingdom. Blood samples were collected at baseline and on day 1, 8, 22, 36 (EOT), 57, 113, and 169 (EOS).

### Outcome measures

The primary endpoint of this study was the safety and tolerability of OVM-200 vaccination, measured by the occurrence and intensity of adverse events. The secondary outcomes comprised: (1) immunogenicity of OVM-200—measured by IFN-ɣ ELISpot to assess T cell responses and anti-survivin IgG ELISA to assess antibody responses; (2) disease progression and tumour response—by evaluating the best overall response (BOR) using Response Evaluation Criteria in Solid Tumour (RECIST) 1.1^20^ at the end of the study; (3) association between immunogenicity measures and clinical response following OVM-200 administration. Phase 1b will further assess these endpoints in the expansion cohort.

### Adverse events

Adverse events (AEs) were evaluated using the Common Terminology Criteria for Adverse Events (CTCAE) version 5.0, in which the severity of AEs were graded 1–5.^21^ AEs were assessed at each visit throughout the treatment period until the end of the study (Week 24/Day 169).

### Anti-survivin IgG assay

Patients’ serum samples were tested for antibody against survivin (anti-survivin antibody) using enzyme-linked immunosorbent assay (ELISA). A 96-well plate was pre-washed. Half of it was subsequently coated with biotinylated recombinant human survivin protein (#SPEC0064) and the other half with assay buffer. After a blocking step, the negative control (NC), positive control (PC) and all samples were serially diluted in assay buffer so the instrument responses would cross the cut-point (with at least one set of responses above and one set of responses below the cut-point).

After incubation, anti-human IgG horseradish peroxidase (HRP) (Abcam, #Ab6759) was loaded, followed by a short incubation period and the addition of tetramethylbenzidine (TMB). The reaction was stopped by adding sulfuric acid and the plate was read on a plate reader at 450 nm. This method measured the total (specific and non-specific) and non-specific binding of the anti-survivin antibodies. Specific binding was calculated by subtracting non-specific binding from the total binding. The anti-survivin antibody titre was reported in 2-fold dilutions starting from 1 in 20. A titre value of <1 in 20 was considered negative.

### PBMC IFN-ɣ assay by ELISpot

Whole blood samples were processed within 24 h of collection to obtain peripheral blood mononuclear cells (PBMCs) for ELISpot analysis. Viable PBMCs were counted using Vi-Cell XR system using the default cell type setting. Samples were considered suitable for analysis if the viability of cells was ≥60%. Cells were added to the assay at a concentration of 2 × 10^5^ cells per well. Cells were treated with: (1) the positive controls: Concanavalin A (Con A) at 0.25 μg/mL (Sigma–Aldrich, #C5275) and anti-CD3 (Mabtech, #3605-1-50) at 100 ng/mL; (2) the test stimulators: overlapping synthetic peptides pool of survivin (Table S1) at 50 μg/mL and OVM-200 at 50 μg/mL; and (3) the background control: the media. Cells were incubated on anti-IFN-ɣ-coated membranes in microtitre plates (Human IFN-γ ELISpotPLUS Kit, MABTECH, #3420-4APT-10). After a wash to remove any unbound biotinylated antibody, Alkaline Phosphatase (ALP) conjugated to streptavidin was added. Any unbound enzymes were removed subsequently by washing and a substrate solution (BCIP/NBT) was added. A blue-black coloured precipitate formed at the site of cytokine localization and appeared as spots, with each individual spot representing an individual IFN-γ secreting cell. These spots were counted using an automated AID ELISpot reader system. The results were reported as the ratio of OVM-200 spot count to background (media) spot count. A robust positive response is a spot count per 10^6^ cells in positive control wells of 4 times the mean of the cell only control.

### Ethical considerations

The study protocol was reviewed and approved by South Central–Oxford A Research Ethics Committee (REC) under the reference 22/SC/0342. The study was conducted in accordance with the Declaration of Helsinki, Good Clinical Practice (GCP) guidelines, and UK Medicines for Human Clinical Use (Clinical Trials) Regulations. All participants have been informed and have provided consent for their data to be included in this study.

### Data presentation, visualization, and statistical analysis

Categorical and continuous variables were reported as absolute numbers, frequencies with percentages, and medians with range (minimum and maximum). The normality of data distribution was tested with Shapiro–Wilk test. Continuous variables were compared between different groups using t-test or Mann–Whitney test as appropriate with the distribution normality. Data visualization and statistical analysis was performed with GraphPad PRISM 9. Results were considered significant if the p-value <0.05.

The comparative statistical tests applied in this phase 1a analysis (t-test and Mann–Whitney test) were not pre-specified in the protocol or statistical analysis plan (SAP), as this phase was primarily exploratory and focused on descriptive safety and immunogenicity outcomes. Prespecified exploratory analyses, such as logistic regression and survival analyses, were defined in the SAP and outlined in the study protocol (Section 7, *Statistical Methods*). These analyses were not performed due to the small sample size (n = 12) and limited statistical power. This sample size reflects the fully accrued phase 1a Cohort in accordance with the predefined 3 + 3 dose-escalation design. Accordingly, only descriptive and non-parametric comparative analyses were conducted. Not performing these exploratory analyses in phase 1a does not constitute a protocol deviation, as the SAP allows for their application only when sufficient data are available. The exploratory analyses will be implemented in the planned phase 1b and phase 2 studies.

### Role of the funding source

The funder of the study had no role in the data collection, data analysis, and data interpretation. The manuscript was written independently by the authors. Although three of the authors (TM, WF, and SJ) are affiliated with Oxford Vacmedix, all data and interpretation were verified by independent investigators and the trial was conducted under Good Clinical Practice guidelines.

## Results

### Vaccine stability

Vaccine stability was assessed at three temperatures: 25 °C, 4 °C and −20 °C. Samples were stored at all three temperatures and analysed for changes against the extensive GMP release criteria. The vaccine showed instability in the form of aggregation into multimeric forms such as trimers and tetramers at 25 °C in a short term (days). This aggregation was lower at 4 °C (weeks). No aggregation was found at −20 °C and annual testing of retained samples of stored at −20 °C for 4 years showed no discernible changes in physical and chemical characteristics.

The survivin ROP vaccine was manufactured under GMP conditions and stored at −20 °C for use in the trial, which represents the standard storage condition for peptide-based vaccines. Reserve undispensed material was held in long term storage at −80 °C.

### Subject characteristics

Overall, twelve (12) patients were vaccinated in this 3 + 3 dose escalation study, in which the 4 doses are grouped in 4 cohorts (Cohort 1: 250 μg, Cohort 2: 500 μg, Cohort 3: 1000 μg, and Cohort 4: 2000 μg). Each cohort includes 3 patients. The age of the patients in the four cohorts ranged from 53 to 74 years, with a median of 63 years. The baseline median anti-survivin IgG titre was <1 in 20 (negative) and the baseline median ELISpot T cell measurement was 33 SFU per million cells. Given the small sample size and exploratory nature of this phase 1a study, the enrolled cohort was not designed to be demographically representative of the broader population of patients with advanced ovarian, prostate, or non-small cell lung cancers. The baseline characteristics of participants are summarised in Table 1.

**Table 1:** Baseline characteristics of participants.

Variables	Median (Min-Max) or n (%)
Age	63 (53–74)
Sex	
Female	9 (75)
Male	3 (25)
Cancer type	
NSCLC	1 (8.3)
Ovarian	8 (66.7)
Prostate	3 (25)
Cancer stage	
Stage 3	1 (8.3)
Stage 4	11 (91.7)
Platelet count, cells/μL	264,500 (192,000–451,000)
Leukocyte count, 10^6^ cells/μL	6060 (2620–12,600)
Absolute neutrophil count, 10^6^ cells/μL	4050 (1560–10,400)
Absolute lymphocyte count, 10^6^ cells/μL	1305 (640–2400)
Neutrophil-to-lymphocyte ratio (NLR)	2.72 (0.83–11.56)
Platelet-to-lymphocyte ratio (PLR)	222.11 (80.00–351.11)
Serum survivin, pg/mL	86.3 (82.46–98.64)
Cancer marker	
CA-125, U/mL (n = 8)	626.00 (339.00–1116.00)
PSA, ng/mL (n = 3)	12.50 (0.01–124.00)
Anti-survivin antibody GMT	<1:20 (<1:20–1:160)
ELISpot T cell (SFU/10^6^ cells)	33 (0–295)

NA, not available; NSCLC, non-small cell lung cancer; SFU, spot-forming units.

### Safety and tolerability

All patients experienced Grade 1 ISRs (Table 2). The reported ISRs included pain, swelling, itching, bruising, muscle aches, erythema, and rash. We did not observe any serious adverse events related to OVM-200: anaphylaxis, angioedema, bronchospasm, systemic rash, nor hypersensitivity reactions.

**Table 2:** Adverse events rate in each cohort.

Adverse events (AEs)	Cohort (n = 3 each), n (%)			
	250 μg	500 μg	1000 μg	2000 μg
**Local/Injection site reactions (ISRs)**				
Grade 1	3 (100)	3 (100)	3 (100)	3 (100)
Grade 2	0 (0)	0 (0)	0 (0)	0 (0)
Grade 3	0 (0)	0 (0)	0 (0)	0 (0)
Grade 4	0 (0)	0 (0)	0 (0)	0 (0)
Grade 5	0 (0)	0 (0)	0 (0)	0 (0)
**Systemic**				
Grade 1	0 (0)	0 (0)	0 (0)	0 (0)
Grade 2	0 (0)	0 (0)	0 (0)	0 (0)
Grade 3	0 (0)	0 (0)	0 (0)	0 (0)
Grade 4	0 (0)	0 (0)	0 (0)	0 (0)
Grade 5	0 (0)	0 (0)	0 (0)	0 (0)

### Immunogenicity and dose selection

**Fig. 3:**
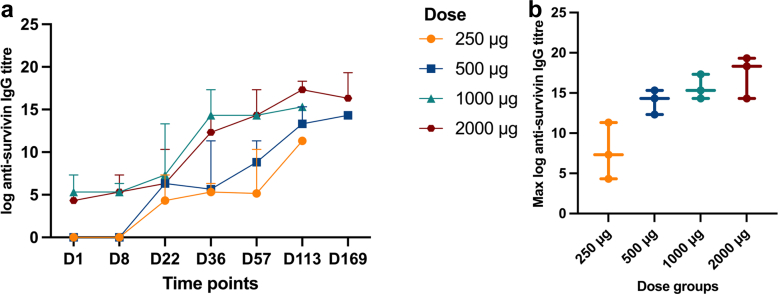
Humoral response following OVM-200 administration, measured by anti-survivin IgG titre: (a) Kinetics of log anti-survivin IgG titre across different time points: day 1, 8, 22, 36, 57, 113, 169. Data are shown as median with range. (b) Maximum log anti-survivin IgG titre achieved in each dose group (250, 500, 1000, 2000 μg). Each symbol represents an individual subject, with bars showing median and range.

**Fig. 4:**
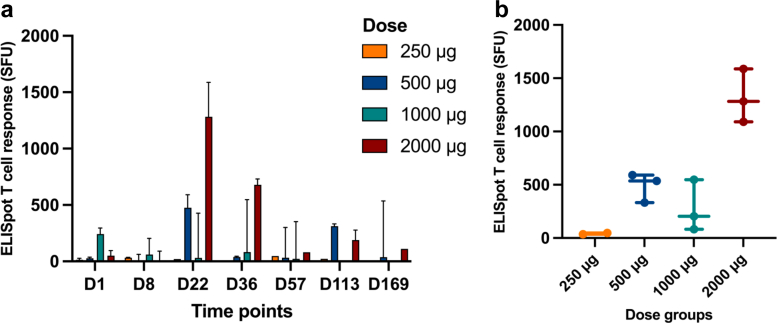
Cellular response following OVM-200 administration, measured by IFN-ɣ ELISpot T cell spot-forming unit (SFU) per 10^6^ cells: (a) Kinetics of T cell response (SFU per 10^6^ cells) across different time points: day 1, 8, 22, 36, 57, 113, 169. Data are shown as median with range. (b) Maximum T cell response (SFU per 10^6^ cells) achieved in each dose group (250, 500, 1000, 2000 μg). Each symbol represents an individual subject, with bars showing median and range.

Cohort 4 demonstrated the best humoral response, based on the anti-survivin antibody titre developed over time and sustained until the end of the study (Fig. 3a, Table S2). Cohort 4 achieved a median anti-survivin IgG titre of 1:327,680 on day 169. In terms of cellular response (Fig. 4, Table S3), cohort 4 exhibited robust positive responses at two time points i.e., a median of 1282 SFU/10^6^ cells on day 22 and 678.5 SFU/10^6^ cells on day 36 (Fig. 4a); however, this response did not last until the end the study. When the maximum anti-survivin IgG titres and ELISpot T-cell responses were compared between the four cohorts, cohort 4 achieved the highest values compared to the other cohorts (Figs. 3b and 4b).

### Disease progression and tumour response

**Fig. 5:**
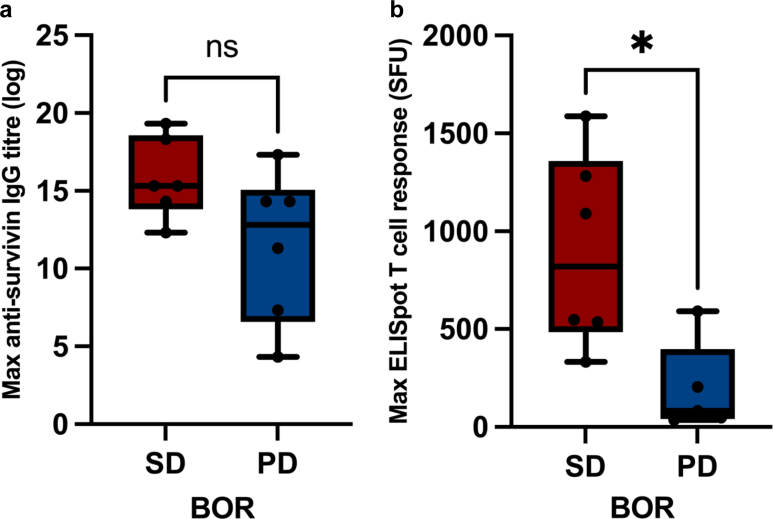
Immunogenic responses stratified by best overall response (BOR): (a) Maximum anti-survivin IgG titre (log-transformed) measured by ELISA and (b) maximum T cell response measured by IFN-γ ELISpot in patients with stable disease (SD, red) vs progressive disease (PD, blue). Each dot represents an individual patient, boxplots show median with interquartile range (IQR), and whiskers indicate the minimum and maximum values. Statistical comparisons were performed Mann–Whitney U test; ns, not significant, ∗p < 0.05.

No patients had a complete or partial response. Six of 12 (50%) patients in Phase 1a achieved best overall responses (BORs) of stable disease (SD) with the remainder having progression (PD) (Table 3). All patients (3 of 3 or 100%) in Cohort 4 had the BORs of SD. Patients who achieved the BOR of SD mounted significantly higher median maximum ELISpot T cell response (SFU per million cell) compared to those with PD (819 vs 83, p = 0.0303) (Fig. 5b). No statistically significant difference in maximum anti-survivin IgG titres was observed between patients with SD and those with PD (15.32 vs 12.82, p = 0.0823; Fig. 5a).

**Table 3:** Best overall responses (BORs) and immunogenic responses based on cohort.

Subjects	Age	Sex	Cancer type	Stage	BOR	Max anti-survivin antibody titre	Max ELISpot T-cell (SFU/10^6^ cells)
**Cohort 1 (250 μg)**							
Patient 1	62	F	Ovarian	4	PD	1 in 2560	47
Patient 2	74	F	Ovarian	4	PD	1 in 20	NA
Patient 3	64	F	Ovarian	4	PD	1 in 160	35
**Cohort 2 (500 μg)**							
Patient 4	68	F	Ovarian	3	SD	1 in 40,960	535
Patient 5	73	M	Prostate	4	SD	1 in 5120	333
Patient 6	53	F	NSCLC	4	PD	1 in 20,480	590
**Cohort 3 (1000 μg)**							
Patient 7	65	F	Ovarian	4	PD	1 in 20,480	83
Patient 8	63	F	Ovarian	4	SD	1 in 40,960	548
Patient 9	58	F	Ovarian	4	PD	1 in 163,840	204
**Cohort 4 (2000 μg)**							
Patient 10	53	F	Ovarian	4	SD	1 in 20,480	1282
Patient 11	59	M	Prostate	4	SD	1 in 655,360	1588
Patient 12	74	M	Prostate	4	SD	1 in 327,680	1090

SFU, spot-forming units.

## Discussion

This phase 1a, multicentre, open-label, non-randomized trial (OVM-200-100) aimed to evaluate the safety, tolerability, immunogenicity, and tumour response of OVM-200, a recombinant overlapping peptides (ROPs)-based therapeutic vaccine derived from survivin, an inhibitor of apoptosis protein highly expressed in various cancers. This study included patients with locally advanced or metastatic NSCLC, ovarian cancer, and prostate cancer who had exhausted standard treatment options or were in a treatment-free interval. Moreover, in contrast to other survivin peptide vaccines,^13^, ^14^, ^15^, ^16^, ^17^^,^^22^^,^^23^ patient selection was not restricted to certain HLA haplotypes. Our findings demonstrate that OVM-200 was well-tolerated, induced a robust humoral response, and a robust though transient T-cell response after 3 doses of OVM-200, showing a dual mode of action for the vaccine. There is some evidence that patients with stable disease had stronger immune response than those with progressive disease but patient follow-up was restricted to 6 months in this phase 1a study.

Overall, OVM-200 was well tolerated, with no serious adverse drug reactions or dose-limiting toxicities (DLTs). The most commonly reported adverse events (AEs) were injection site reactions (ISRs), all of which were Grade 1. These findings are consistent with previous phase 1 studies of survivin-derived peptide vaccines in cancer patients which have generally demonstrated favourable safety profiles ranging from none to mild or moderate reactions.^14^^,^^22^^,^^23^ The acceptable safety profile of OVM-200 supports further evaluation in the subsequent phase of clinical trial.

The preliminary immunogenicity of OVM-200 in the phase 1a dose-escalation study was assessed to select the dose for the dose-expansion (phase 1b) study. The immunogenicity was measured by anti-survivin antibody titre for the humoral response and PBMC IFN-ɣ ELISpot for the cellular response. The 2000 μg dose group exhibited the highest mean anti-survivin antibody titre (1:131,072) by the end of the study (day 169), indicating a strong humoral response. The antibody response increased significantly over time, particularly after the final dose on Day 36, and remained elevated until the end-of-study visit (Day 169). This suggests that OVM-200 successfully induces a sustained B-cell-mediated immune response. This finding aligns with a previous study of a survivin long peptide vaccine (SurVaxM) in patients with recurrent malignant glioma, in which high titres of antibodies to survivin epitopes and wild-type survivin were developed.^16^

In this study, cohort 4 also achieved a robust cellular response although it did not last as long as the humoral response. Cohort 4 (2000 μg dose group) showed robust T-cell responses on days 22 and 36; this response did not remain as high at the end of the study. The magnitude and short-lived durability of the cellular immune response in this study is comparable to that observed in a neoantigen clinical trial^24^ even though different assay methods were employed. In our study, the immune responses were assessed using ELISpot and ELISA, which are appropriate clinical tools to demonstrate vaccine immunogenicity and safety. We note that more advanced techniques, such as TCR clonotype tracking as used by Rojas et al.,^24^ provide valuable mechanistic information on clonal expansion and persistence, but are not typically required at this stage of development. This pattern is also consistent with findings from other survivin peptide vaccine studies.^14^, ^15^, ^16^^,^^22^^,^^23^ For the phase 1b trial, the MHRA has approved the administration of up to 11 OVM-200 doses. This is anticipated to lead to a more sustained T-cell response.

As expected in this population with advanced cancers that had exhausted standard therapies, there were no complete or partial responses. Some patients had extended periods of stable disease but this is difficult to interpret given the heterogeneity of the cancer types studied and the small size of phase 1a. We conducted preliminary statistical comparisons of the immunogenic responses (IgG titre and T cell response) between patients who achieved stable disease and those with progressive disease as the best overall response. Patients with SD mounted stronger immune responses, with the difference being most pronounced and statistically significant for T cell responses as measured by IFN-γ ELISpot (SFU per million cells). However, these findings should be considered preliminary as the current analysis is limited by the small sample size and short follow-up. The clinical efficacy will be better assessed in the next part (phase 1b) of the study, which incorporates a larger population of patients and a longer maximum treatment duration. Previous studies demonstrated that survivin-based peptide vaccination could yield disease stabilisation^16^^,^^23^ and significant improvements in overall and progression-free survival.^16^ However, it is important to note that our phase 1a trial involved patients with advanced cancer who had already exhausted standard therapies, whereas the majority of patients in one of the previous studies^16^ were at earlier stages of the disease.

Survivin-based vaccines have been explored in multiple clinical settings.^14^, ^15^, ^16^^,^^22^^,^^23^ SurVaxM, also a survivin peptide vaccine, demonstrated safety and preliminary efficacy in phase 1 trials for glioblastoma^16^ and neuroendocrine tumours,^15^ though its immunogenicity was limited to specific HLA haplotypes (HLA-A∗02, -A∗03, -A∗11, or -A∗24). Unlike SurVaxM, OVM-200 is designed to induce an immune response independent of HLA haplotype, which could broaden the applicability across diverse patient populations. Trials from both vaccines have shown limitations in a sustained T-cell response. Combination approaches that promote durable T cell responses should be explored in future trials. These strategies include combination with immunostimulants, immune checkpoint blockade,^25^ or cytokine therapies that support T-cell survival and memory development (e.g. IL-15, IL-7, and new-engineered IL-2).^26^^,^^27^

Peptide-based cancer vaccines, including the ones derived from survivin, have shown promising immunogenicity but modest clinical efficacy as monotherapies.^28^ This is likely due to several factors, such as the identification of specific target tumour antigens, the limited immunogenicity of peptides, and a highly immunosuppressive tumour microenvironment (TME).^28^^,^^29^ Cancer cells also employ immune evasion mechanisms, such as the upregulation of immune checkpoints and downregulation of co-stimulatory molecules.^30^

This phase 1a study has several limitations. The small sample size and heterogeneity of tumour types limit the statistical power and generalisability of the findings. The study was not designed to be demographically representative of the broader cancer population, as its primary aim was to assess safety and preliminary immunogenicity. The open-label design and absence of a control arm limit causal inference regarding the observed clinical outcomes. Additionally, the follow-up duration was relatively short, which restricted assessment of long-term immune persistence and clinical benefit. More advanced immunophenotyping techniques, such as flow cytometry and T-cell receptor sequencing, were not included at this stage but are planned for the subsequent phases. These next-phase trials, with larger and more homogeneous cohorts, extended dosing, and comprehensive immune monitoring, will allow for more definitive evaluation of safety, efficacy, and mechanistic correlates.

Given the favourable safety profile and immunogenicity of OVM-200 in this phase 1a trial, further evaluation in a phase 1b trial should be conducted using the dose of 2000 μg, with a longer follow-up duration and larger sample size if feasible. The ongoing phase 1b trial will further evaluate the safety/tolerability and immunogenicity of OVM-200 at the 2000 μg dose, as well as the impact of the approved booster doses.

This phase 1a study demonstrates that OVM-200 is well tolerated and induces a robust humoral response, with a considerable cellular response. Phase 1b is ongoing to further evaluate its safety and efficacy at the selected dose, as well as the impact of extended dosing over 6 months.

## Contributors

WW and SJ wrote the first draft of the manuscript. WW, TM, and SJ contributed to the data curation. WW undertook the formal data analysis and visualisation. WW, TM, WF, and SJ had full access to all the data in the study. TM, WF, and SJ verified the data in the study and were responsible in the supervision of the trial. MF, MT, FT, and AW were responsible for the recruitment and management of the patients in their respective trial site. All authors contributed to data interpretation, drafting and review of the final manuscript, and had final responsibility for the decision to submit for publication.

## Data sharing statement

Individual patient data from this trial will not be published in the public domain. Requests for data sharing will be reviewed on a case-by-case basis and subject to data sharing agreements.

## Declaration of interests

TM is the Chief Medical Officer of Oxford Vacmedix. SJ is the founder and Chief Scientific Officer of Oxford Vacmedix. WF is the Chief Executive Officer of Oxford Vacmedix. MDF acknowledges research funding from AstraZeneca, Boehringer Ingelheim, MSD and Merck; advisory board membership for Transgene; Honoraria for consulting or conference attendance with Achilles, Amgen, AstraZeneca, Bayer, Boxer, Bristol Myers Squibb, Celgene, EQRx, Guardant Health, Immutep, Ixogen, Janssen, Merck, MSD, Nanobiotix, Novartis, Oxford VacMedix, Pharmamar, Pfizer, Roche, Takeda and UltraHuman; and support by the UCL/UCLH NIHR Biomedical Research Centre, delivering early phase trials within the UCLH NIHR Clinical Research Facility supported by the UCL Experimental Cancer Medicine Centre. All other authors declare no competing interests.

## Appendix A Supplementary data

Protocol

Statistical Analysis Plan
