# Supplementary material for: Survivin recombinant overlapping peptide (ROP) vaccine in advanced solid tumours: a first-in-human, multicentre, open-label, phase 1a dose-escalation study
Source: eClinicalMedicine. 2025 Dec 27;91:103717. doi: 10.1016/j.eclinm.2025.103717 (PMC12796589; doi:10.1016/j.eclinm.2025.103717)
Supplement: Protocol [file mmc1.pdf]

Protocol

A Phase 1, Multicentre, Open-label, Nonrandomised, First-in-human Study of OVM-200 as a Therapeutic Vaccine in Patients with Locally Advanced or Metastatic Non-Small Cell Lung Cancer, Ovarian Cancer, and Prostate Cancer

Protocol Status: Final
Protocol Date: 5 May 2021
Protocol Version: 1.0

Investigational Medicinal Product: OVM-200

Protocol Reference Number: OVM-200-100
EudraCT Number: 2021-001545-12

Sponsor:
Oxford Vacmedix Ltd.
The Magdalen Centre
Oxford Science Park
Oxford, OX4 4GA United Kingdom

Multiple Sites

Sponsor Signatory:
Thomas Morris, BSc, MB BCh, LlM, MRCP FFPM

Medical Monitor:
Stefanella Bortini, MD, PhD

Information described herein is confidential and may be disclosed only with the express written permission of the sponsor.

SPONSOR APPROVAL

I have read the protocol and approve it:

5th May 2021

Thomas Morris, BSc, MB BCh, LlM, MRCP FFPM
Chief Medical Officer

Date

INVESTIGATOR AGREEMENT

I have read the protocol and agree to conduct the study as described herein.

____
Investigator’s Name (Printed)

Investigator’s Signature	Date

SYNOPSIS

Study Title

A Phase 1, Multicentre, Open-label, Nonrandomised, First-in-human Study of OVM-200 as a Therapeutic Vaccine in Patients with Locally Advanced or Metastatic Non-Small Cell Lung Cancer, Ovarian Cancer, and Prostate Cancer

Objectives

The primary objective of the study is:

- To characterise the safety and tolerability of OVM-200 in patients with tumour types known to over-express survivin (non-small cell lung cancer [NSCLC], ovarian cancer, and prostate cancer).

The secondary objectives of the study are:

- To determine the immune response following administration of OVM-200 in patients with tumour types known to over-express survivin.
- To determine the recommended dose of OVM-200 for further development in patients with tumour types known to over-express survivin.

The exploratory objectives of the study are:

- To conduct a preliminary exploration of the antitumour activity of OVM-200 in patients with tumour types known to over-express survivin.
- To evaluate the correlation of survivin expression with response to the administration of OVM-200.

Study Design

This is a 2-part study in patients with:

i. NSCLC
ii. ovarian cancer, and
iii. prostate cancer.

The first part (Phase 1a) comprises a first-in-human (FIH) multiple-dose, sequential-cohort 3+3 design to establish a dose of OVM-200 that is safe and tolerable, and that elicits an immune response in humans.

This dose will be taken forward into the second part (Phase 1b) of the study. Phase 1b will further assess the safety and tolerability of the selected dose and investigate the immune and tumour response in 3 expansion cohorts of additional patients with NSCLC, ovarian cancer, and prostate cancer.

A schedule of assessments is available in Appendix 6.

Phase 1a

OVM-200 will be administered after at least 1 line of systemic cancer treatment is completed, with the exception of supportive therapies and androgen deprivation therapies for prostate cancer patients, which may be continued throughout the study.

After a screening period of up to 21 days, eligible patients will arrive at the site on the first day of dosing (Day 1) to confirm eligibility, complete predose baseline assessments, and receive the first dose of OVM-200. Patients will receive 3 doses of OVM-200 at 2-week intervals. The patients will remain at the site for at least 4 hours after each dose for observation. Between dose administrations, patients will return for weekly visits through the end-of-treatment (EOT) visit on Week 5. There will be additional visits on Weeks 8 and 16 and an end-of-study (EOS) visit on Week 24.

A safety review committee (SRC) will review all available cumulative safety and immune response data throughout the trial.

Phase 1a will be a standard 3+3 design with up to 4 cohorts.

Three patients will initially be enrolled into a given dose cohort. A single sentinel patient will be dosed initially with 2 additional patients dosed at the same dose level at least 2 weeks later, if no dose-limiting toxicities (DLTs) or other safety and tolerability concerns are observed in the sentinel patient. If a DLT or other notable safety event is experienced in the sentinel patient, the SRC will review all available data before deciding on the continuation of dosing in subsequent patients.

If no DLT is observed in any of these patients to week 5 (1 week after the final vaccination in the last patient to be dosed) dosing will commence in the next cohort. If one patient develops a DLT at a specific dose, an additional three patients will be enrolled into that same dose cohort. Development of DLTs in more than 1 of 6 patients in a specific dose cohort suggests that the maximum tolerated dose (MTD) has been exceeded, and further dose escalation will not be pursued.

In addition, cohort 4 will not proceed if a clear plateau in immune response is observed in the previous 3 cohorts.

The SRC will review data from Phase 1a and make a recommendation on the Phase 1b dose based on safety and immune response. The dose in Phase 1b will not exceed the dose safely administered in Phase 1a.

Phase 1b

OVM-200 will be administered after at least 1 line of systemic cancer treatment is completed, with the exception of supportive therapies and androgen deprivation therapies for prostate cancer patients, which may be continued throughout the study.

After a screening period of up to 21 days, eligible patients will arrive at the site on the first day of dosing (Day 1) to confirm eligibility, complete predose baseline assessments, and receive the first dose of OVM-200. Patients will receive 3 doses of OVM-200 at 2-week intervals. Patients will remain at the site for at least 2 hours after each dose for observation.

Between dose administrations, patients will return for weekly visits through the EOT visit on Week 5. There will be additional visits on Weeks 8 and 16 and an EOS visit on Week 24.

Number of Patients

Phase 1a: 3 to 24 patients will be studied in 1 to 4 dose cohorts of 3-6 patients each.

Phase 1b: A minimum of 24 patients will be studied in 3 therapeutic indication cohorts of approximately 8 patients each. More than 8 patients may be enrolled in a cohort, for a maximum of 28 patients total in this phase of the study. If more than 8 patients are enrolled in 1 of the cohorts (up to a maximum of 10 patients), fewer patients may be enrolled in either or both of the other cohorts.

Diagnosis and Main Criteria for Inclusion

Male and female patients with metastatic or locally advanced inoperable NSCLC, ovarian cancer, or prostate cancer that have already received at least 1 line of approved cancer therapy and are currently, in the opinion of the investigator, considered unlikely to require further cancer therapy up to the Week 8 assessment timepoint. Suitable patients may be those that have exhausted all currently approved therapies or, are considered not to require further lines of cancer therapy as they have no evidence of progressive disease after completing a prior line of cancer therapy or, in the case of prostate cancer patients, have no evidence of progressive disease while on approved anti-hormonal therapy (LHRH analogues, androgen receptor antagonists or an androgen synthesis inhibitor). Patients between 18 and 75 years of age, inclusive, with an Eastern Cooperative Oncology Group (ECOG, Section 7.2.6) performance status ≤ 2 and adequate bone marrow, renal, and hepatic functions will be eligible for enrolment in the study.

Investigational Medicinal Products, Dose, and Mode of Administration

Test product: 2 mg/mL OVM-200 solution

Proposed dose levels for Phase 1a: 250, 500, and 1000 µg. The planned doses may be adjusted based on SRC recommendations. Following review of the data, 1 additional dose level may be added up to a maximum of 2000 µg.

The dose level for Phase 1b will be selected following review of the data from Phase 1a and will not exceed the dose safely administered in Phase 1a.

Three doses of OVM-200 plus adjuvant will be injected subcutaneously (SC) at 2-week intervals.

Duration of Patient Participation in the Study

Planned Screening duration: approximately 3 weeks.

Planned study duration (screening to EOS visit): up to approximately 190 days.

Endpoints

Safety

The primary endpoint (safety and tolerability) for this study is the occurrence and intensity of adverse events.

Efficacy

The secondary endpoint (efficacy) is the immune response to OVM-200 as measured by enzyme-linked immune absorbent spot (ELISpot) for T-cell responses and enzyme-linked immunosorbent assay (ELISA) for antibody responses. All assays have been fully validated for use in this clinical study.

The exploratory efficacy endpoints are as follows:

- Disease progression and tumour response evaluated using Response Evaluation Criteria in Solid Tumour (RECIST) v1.1 including objective response rate (ORR) over 24 weeks, time to response (TTR), duration of response (DoR), progression-free survival (PFS), and overall survival (OS), as appropriate. Bone lesions in prostate cancer patients will be evaluated using the methodologies described by the Prostate Cancer Working Group 3 (PCWG3).
- In ovarian cancer patients, tumour marker cancer antigen-125 (CA-125) measured and evaluated according to Gynecologic Cancer Intergroup Criteria (GCIC).
- In prostate cancer patients, tumour markers prostate-specific antigen (PSA) and total alkaline phosphatase (ALP) as per PCWG3 response criteria.
- Survivin expression will be correlated to response (based on immune response, tumour assessments, and tumour marker measurements) achieved after OVM-200 administration.

Statistical Methods

Study Populations

The safety evaluable population is defined as all patients in either phase who received OVM-200 regardless of the duration of treatment received. Safety parameters and efficacy parameters such as PFS; OS; and CA-125, PSA, and ALP responses will be assessed in this population.

The immune response evaluable population is defined as all patients who received OVM-200 in either Phase and had at least 1 pre-dose and 1 postbaseline sample taken to assess immune response. Immune response as measured by ELISpot and ELISA will be assessed in this population.

The RECIST response evaluable population is defined as all patients who received OVM-200 in either Phase and have at least 1 postbaseline assessment for the RECIST response. The ORR, TTR, and DoR will be assessed in this population.

Safety

Safety data will be summarised using descriptive statistics. Adverse event terms will be coded using Medical Dictionary for Drug Regulatory Activities (MedDRA) Version 24.0 and summarised by body system, preferred term, severity, and relationship to study drug. Clinical laboratory assessments will be converted to standard units and summarised by maximum National Cancer Institute Common Terminology Criteria for Adverse Events v5.0 severity grade. Vital signs and 12-lead electrocardiograms will be summarised using descriptive statistics.

Efficacy

Immune Response

In Phase 1a, immune response will be summarised using frequency counts and percentages. Immune response will be presented by therapeutic indication and overall using methodology akin to that used in basket study designs (1). Immune response may also be analysed supportively in a Bayesian manner by combination of responses in Phases 1a and 1b.

Disease Progression and Tumour Response

The ORR is defined as patients with an investigator-assessed response of complete response (CR) or partial response (PR) as per RECIST. In Phase 1a, ORR will be summarised by visit and dose using frequency counts and percentages. The ORR may be analysed supportively in a Bayesian manner by combination of responses in Phases 1a and 1b.

The DoR is defined for patients with an investigator-assessed response of CR or PR as the time from the date of objective response to the first of disease progression (bone or soft tissue) or death. Patients who do not experience disease progression or death will be censored at the time of their last RECIST assessment. Kaplan-Meier plots will be generated for DoR if 5 or more patients in Phases 1a and 1b combined experience disease progression or death.

The TTR is defined for patients with CR or PR as the time from the first dose of OVM-200 to the first objective response (CR or PR). The OS is defined as the time from study entry to death due to any cause. The PFS is defined as the time from study entry to disease progression (bone or soft tissue) or death. Censoring at the time of the last RECIST assessment will occur for TTR for patients who do not experience CR or PR and for PFS for patients who do not experience disease progression or death. Patients who are alive will be censored at the time of their last site contact for OS. The TTR, OS, and PFS may be displayed graphically using Kaplan-Meier plots utilising the combined data from Phases 1a and 1b.

Cancer Antigen-125 Response

In ovarian cancer patients, CA-125 response will be based on the GCIC. The CA-125 response will be summarised by visit using frequency counts and percentages. The CA-125 measures over time will be displayed graphically for each patient and maximal change based on the overall visit responses will be tabulated and displayed using a waterfall plot.

Prostate-specific Antigen and Total Alkaline Phosphatase Response

In prostate cancer patients, the change from baseline in PSA and ALP on the ratio scale will be tabulated by visit, and the maximal change based on the overall visit assessments will be tabulated and displayed using a waterfall plot. The PSA and ALP response will be tabulated by visit using frequency counts and percentages. An estimated PSA doubling time (PSADT) before study entry will be determined and tabulated with estimated on-study PSADT. Longitudinal PSA measures over time will be displayed graphically for each patient.

Relationship to Survivin Expression

The relationship between survivin expression and immune response, objective tumour response (based on RECIST), and tumour marker response (CA-125 [ovarian cancer patients], and PSA and ALP [prostate cancer patients]) will be explored via logistic regression analyses. The log (postdose/baseline) survivin expression will be included as the independent variable in logistic regression analyses. For PSA and ALP, the log odds of response will be characterised as a function of log(post/baseline) expression level.

Cox proportional hazards model may be used to explore the relationship between TTR, DoR, OS, and PFS and baseline survivin expression.

TABLE OF CONTENTS

TITLE PAGE 1
SPONSOR APPROVAL 2
INVESTIGATOR AGREEMENT 3
SYNOPSIS 4
TABLE OF CONTENTS 10
LIST OF TABLES 12
LIST OF FIGURES 12
LIST OF ABBREVIATIONS 13
1. INTRODUCTION 15
1.1. Overview and Mechanism of Action for OVM-200 15
1.1.1. Survivin 15
1.1.2. Mechanism of Action 15
1.1.3. Adjuvant (Montanide) 16
1.2. Summary of Nonclinical Pharmacology 17
1.3. Summary of Toxicology 18
1.4. Summary of Nonclinical Pharmacokinetics 19
1.5. Summary of Clinical Experience 19
1.6. Study Rationale 19
1.7. Benefit-risk Assessment 19
1.7.1. Potential Risks 20
1.7.2. Potential Benefits 20
1.7.3. Overall Risk-benefit Assessment 20
2. OBJECTIVES AND ENDPOINTS 22
2.1. Objectives 22
2.2. Endpoints 22
2.2.1. Safety Endpoint 22
2.2.2. Efficacy Endpoints 22
3. INVESTIGATIONAL PLAN 23
3.1. Overall Study Design and Plan 23
3.1.1. Phase 1a 25
3.1.2. Criteria for Transition to Phase 1b 26
3.1.3. Phase 1b 26
3.2. Study Start and End of Study Definitions 27
3.3. Discussion of Study Design 27
3.3.1. Dose Interval 28
3.4. Selection of Doses in the Study 28
3.5. Dose Escalation 29

3.6. Dose-limiting Toxicities 30
3.7. Stopping rules 31
3.7.1. Individual patient (Phase 1a and 1b)....31
3.7.2. Phase 1a 31
3.7.3. Progression to Phase 1b 31
3.7.4. Phase 1b 31
3.7.5. Termination of the study....31
4. SELECTION OF STUDY POPULATION 31
4.1. Inclusion Criteria 31
4.2. Exclusion Criteria 33
4.3. Patient Number and Identification....34
4.4. Patient Withdrawal and Replacement....34
4.5. Study Termination 35
5. STUDY TREATMENTS....35
5.1. Description, Storage, Packaging, and Labelling....35
5.2. Study Treatment Administration....36
5.2.1. Dose Modification 36
5.3. Randomisation 36
5.4. Blinding....36
5.5. Treatment Compliance....36
5.6. Drug Accountability....36
6. CONCOMITANT THERAPIES AND OTHER RESTRICTIONS 37
6.1. Permitted Concomitant Therapies....37
6.2. Restricted Concomitant Therapies....37
7. STUDY ASSESSMENTS AND PROCEDURES....38
7.1. Efficacy Assessments....38
7.1.1. Immune Response....38
7.1.2. Tumour Response 38
7.1.3. Tumour Markers 39
7.1.4. Tumour Survivin Expression 39
7.2. Safety and Tolerability Assessments 39
7.2.1. Adverse Events 39
7.2.2. Clinical Laboratory Evaluations 41
7.2.3. Vital Signs....41
7.2.4. Electrocardiogram....42
7.2.5. Physical Examination....42
7.2.6. Eastern Cooperative Oncology Group (ECOG) Performance Score....42
8. SAMPLE SIZE AND DATA ANALYSIS....43
8.1. Determination of Sample Size 43
8.2. Analysis Populations....43

8.2.1. Safety Evaluable Population 43
8.2.2. Immune Response Evaluable Population 43
8.2.3. Response Evaluation Criteria in Solid Tumour Response Evaluable Population 43
8.3. General Considerations 43
8.4. Efficacy Analyses 43
8.4.1. Immune Response 43
8.4.2. Objective Response Rate 44
8.4.3. Time to Response 44
8.4.4. Duration of Response 44
8.4.5. Overall Survival 44
8.4.6. Progression-free Survival 44
8.4.7. Cancer Antigen-125 Response 45
8.4.8. Prostate-specific Antigen and Total Alkaline Phosphatase Response 45
8.4.9. Relationship to Survivin Expression 45
8.5. Safety Analysis 46
8.6. Interim Analysis 46
9. REFERENCES 47
10. APPENDICES 50
Appendix 1: Response Evaluation Criteria in Solid Tumours v1.1 51
Appendix 2: Adverse Event Reporting 54
Appendix 3: Clinical Laboratory Evaluations 58
Appendix 4: Contraception Guidance 59
Appendix 5: Regulatory, Ethical, and Study Oversight Considerations 62
Appendix 6: Schedule of Assessments 65

LIST OF TABLES

Table 1: Expression of Survivin Protein 28
Table 2: Proposed Investigational Medicinal Product Dose Levels for Phase 1a 30
Table 3: Eastern Cooperative Oncology Group Performance Status Scale 42

LIST OF FIGURES

Figure 1: Schematic of ROPs 16
Figure 2: Study Schematic 24
Figure 3: Planned Dose Levels (Phase 1a) 26

LIST OF ABBREVIATIONS

Abbreviation	Definition
ADA	antidrug antibody
ADL	activities of daily living
AE	adverse event
ALP	total alkaline phosphatase
BMI	body mass index
CA-125	cancer antigen-125
CFR	Code of Federal Regulations
CI	confidence interval
CR	complete response
CRO	Contract Research Organisation
CT	computed tomography
CTL	cytotoxic T lymphocytes
DoR	duration of response
DLT	dose-limiting toxicity
EC	ethics committee
ECG	electrocardiogram
ECOG	Eastern Cooperative Oncology Group
eCRF	electronic case report form
EDC	electronic data capture
ELISA	enzyme-linked immunosorbent assay
ELISpot	enzyme-linked immune absorbent spot
EOS	end of study
EOT	end of treatment
FIH	first-in-human
GCIC	Gynecologic Cancer Intergroup Criteria
GCP	Good Clinical Practice
GLP	Good Laboratory Practice
HCV	hepatitis C virus
HIV	human immunodeficiency virus
IAP	inhibitor of apoptosis
IB	investigator’s brochure
ICF	informed consent form
ICH	International Council for/Conference on Harmonisation
IFN	interferon
IMP	Investigational Medicinal Product
MedDRA	Medical Dictionary for Drug Regulatory Activities

MHC	Major Histocompatibility
MPLA	monophosphoryl lipid A
MRI	magnetic resonance imaging
NCI CTCAE	National Cancer Institute Common Terminology Criteria for Adverse Events
NSCLC	Non-small cell lung cancer
ORR	objective response rate
OS	overall survival
PCWG3	Prostate Cancer Working Group 3
PD	progressive disease
PFS	progression-free survival
PR	partial response
PSA	prostate-specific antigen
PSADT	prostate-specific antigen doubling time
PSS	Patient Safety Solutions
QTcF	QT interval corrected for heart rate using Fridericia's method
RECIST	Response Evaluation Criteria in Solid Tumour
ROP	recombinant overlapping peptide
SAE	serious adverse event
SC	subcutaneous
SD	stable disease
SRC	safety review committee
TMF	trial master file
TTR	time to response

1. INTRODUCTION

1.1. Overview and Mechanism of Action for OVM-200

OVM-200 is being developed as a therapeutic cancer vaccine targeting survivin, by Oxford Vacmedix Ltd., hereafter referred to as “OVM”.

OVM-200 is a recombinant overlapping peptide (ROP), which for administration, is combined with the adjuvant Montanide ISA 51 VG, hereafter referred to as “Montanide”. Full details can be found in separate investigator’s brochures (IBs) for OVM-200 and Montanide, respectively.

1.1.1. Survivin

Survivin is a member of the inhibitor of apoptosis (IAP) family. IAPs act downstream of a broad range of stimuli, such as cytokines and extracellular matrix interactions, to regulate cell survival, proliferation and migration. These processes are dysregulated during tumour generation and are critical to the metastatic spread of the disease. Survivin is expressed in a variety of apoptosis-regulated organs during embryonic and foetal development, but is undetectable in most normal, terminally differentiated cells in adults. However, IAPs, including survivin, are commonly upregulated in cancer. The survivin protein inhibits caspase activation, thereby leading to negative regulation of apoptosis, or programmed cell death (2, 3).

There is a potential role for survivin in regulating function in normal adult-cells, particularly vascular endothelial cells, polymorphonuclear cells, T-cells, erythroid, and hematopoietic progenitor cells. As a largely intracellular protein, survivin is degraded by the proteasome. Resulting epitopes are presented on the surface of tumour cells by Major Histocompatibility (MHC) class I molecules. Accordingly, survivin-specific cytotoxic T lymphocytes (CTL) have been identified in cancer patients. In addition to T-cell-mediated immunity, many cancer patients develop humoral immune responses to survivin with anti-survivin antibodies detectable in serum (2, 3).

Therefore, survivin is potentially immunogenic, which could provide the basis for induction of therapeutic anti-tumour immunity in cancer patients in whom the immune system is already primed to recognise survivin.

1.1.2. Mechanism of Action

The ROP, OVM-200, is an artificial protein composed of overlapping peptides taken from the target protein survivin interspersed by the target sequence (LRMK) for cathepsin S, a protease found in the endosomes of antigen presenting cells (APCs), including dendritic cells (DCs) and macrophages.

Administration of OVM-200 leads to endosomal uptake into DCs, where OVM-200 is processed, ie cleaved by cathepsin S, into individual peptides (as shown in Figure 1). These peptides are cross presented on MHC class I molecules efficiently, even in CD4+ cell depleted individuals (4).

Figure 1: Schematic of ROPs

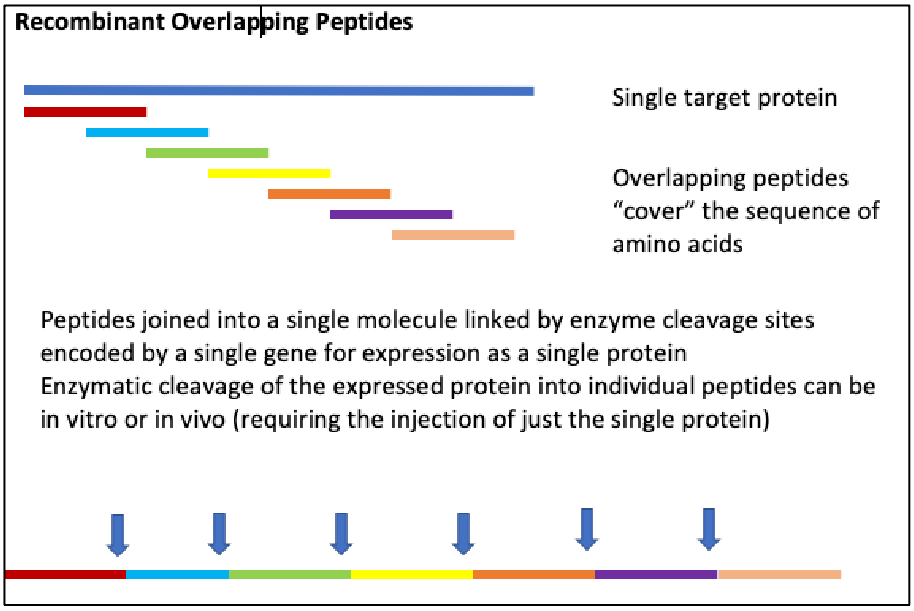

The exogenously applied pool of overlapping peptides can stimulate CD4+ and CD8+ cell immunity to a potentially clinically significant level (4). Therefore, a ROP is suggested to be more effective than the native protein in antigen presentation.

There are two forms of adaptive immune responses by which a cancer vaccine can mediate an effect:

- Humoral immune response - production of antibodies
- Cellular immune response - production of helper T-cells (CD4+ cells) and cytotoxic T-cells (CD8+ cells).

Both types of T-cells are important for supressing tumour cells. However, vaccines that work predominantly through the generation of CD8+ cells have been found to be more effective in destroying tumour cells (5). Conventional sub-unit vaccines are based on full-sized proteins produced by a tumour, which primarily result in the generation of antibodies and CD4+ cells, rather than CD8+ cells (6). In contrast, the ROP, OVM-200, mediates its effects through the production of CD8+ cells, in addition to CD4+ cells and antibodies (4, 7). Therefore, OVM-200 may be more effective in targeting tumour cells than whole protein vaccine approaches.

1.1.3. Adjuvant (Montanide)

An adjuvant augments the anti-tumour immune response by mediating stimulatory effects on anti-tumour T-cell responses (8). Montanide is an emulsion that creates a depot effect resulting in slow release of the antigen at the injection site. In addition, emulsions such as Montanide result in inflammation and recruitment of antigen presenting cells such as macrophages and lymphocytes (also see separate Montanide IB) thus resulting in an increased immune response to an antigen.

1.2. Summary of Nonclinical Pharmacology

The potential for OVM-200 to be effective in the treatment of cancer has been investigated in a series of non-clinical pharmacology models. To confirm that B16-survivin tumour growth can be significantly inhibited in OVM-200 prevaccinated mice, C57BL/6 mice were administered SC vaccinations of 100 µg OVM-200, 100 µg survivin, or control (phosphate buffered saline [PBS]) with monophosphoryl lipid A (MPLA) adjuvant on days 0, 21 and 42. Three weeks after the last vaccination, mice were inoculated with B16-survivin cells. Tumours of OVM-200 prevaccinated mice grew slower than survivin prevaccinated mice, resulting in significantly smaller tumours in the OVM-200 prevaccinated mice than the survivin, or PBS prevaccinated mice (measured 21 days after tumour cell inoculation). Prevaccination with OVM-200 also significantly extended overall survival (OS) in comparison to survivin or PBS.

To investigate OVM-200 as a therapeutic vaccine, B16-survivin tumours were inoculated in mice. Following a 5-day period for the tumours to establish, 100 µg OVM-200 with MPLA, control (PBS) with MPLA or PBS alone was administered SC on days 5, 12 and 19. On Day 21, following the final vaccination, the tumour volumes of the OVM-200 vaccinated group were significantly smaller than in the control groups, and vaccination with OVM-200 extended OS in comparison to controls. The levels of Interferon (IFN)-ɣ producing antigen-specific T-cells were evaluated in spleen using a standard enzyme-linked immune absorbent spot (ELISpot) assay. OVM-200 vaccination induced significant levels of IFN-ɣ producing survivin or OVM-200 specific T-cells. The antibody levels for survivin, or OVM-200, in serum were investigated using an enzyme-linked immunosorbent assay (ELISA). Significantly higher antibody levels against both survivin and OVM-200 were detected in OVM-200 vaccinated mice than in the control group.

To evaluate whether the therapeutic efficacy of OVM-200 vaccine could be enhanced by coadministration of immunotherapeutic antibodies, OVM-200 and MPLA was combined with costimulatory TNF-receptor agonist (α4-1BB) antibody. B16-survivin tumours were inoculated in mice, and 100 µg OVM-200 with MPLA was administered SC on days 5, 12 and 19 and α4-1BB antibody was administered intraperitoneally every 3 days for a total of 20 days, both as standalone treatment or in combination. Either α4-1BB antibody or OVM-200 alone was able to delay tumour growth. Significant therapeutic improvements were observed when α4-1BB was administered in combination with OVM-200, which also resulted in a significantly improved survival rate.

As supportive data, to verify that a mouse survivin-derived ROP vaccine can stimulate the immune system of mice, mice were inoculated with 100 µg of an ROP-mSurvivin based on mouse survivin with MPLA every 2 weeks for a total of 4 weeks. The ELISpot assay showed that mice immunised with ROP-mSurvivin had higher specific T-cell responses than MPLA immunised control mice. Vaccination with ROP-mSurvivin also resulted in higher levels of antibodies against both mouse survivin and ROP-mSurvivin than in the control group.

Initial pharmacology studies were conducted with OVM-200 in combination with MPLA. However, due to unforeseen supply issues with MPLA, Montanide will be used as the adjuvant in this study. The data supporting the general activity of Montanide as an adjuvant are provided in the Montanide IB. However, this has also been tested in combination with OVM-200. In a formulation study conducted with Montanide in combination with OVM-200,

a significant immune response to survivin was generated. In a study comparing OVM-200 combined with either Montanide or MPLA, although a slightly lower level of immune response was seen with Montanide, particularly at early timepoints, overall a significant response was seen to both MPLA and Montanide demonstrating that Montanide acts as and adjuvant in combination with OVM-200 and thus is suitable for use in this clinical study.

The totality of the pharmacological evidence supports the potential for OVM-200 in combination with either Montanide or MPLA to generate an immune response to survivin and consequently slow tumour growth. Data are described in more detail in the OVM-200 IB.

1.3. Summary of Toxicology

An 8-week, repeat-dose, toxicity study of 200 μg OVM-200 with 50 μg 3D (6-acyl) MPLA adjuvant has been completed in mice. OVM-200 was well tolerated when administered SC every 2 weeks. There were treatment-related findings in haematology and blood chemistry and findings in macroscopic and microscopic investigations in the spleen, axillary lymph nodes, and injection sites, but these were not considered adverse. Cytokine activity and antidrug antibody (ADA) response in the peripheral blood and ELISpot IFN-ɣ levels in the spleen showed a positive immune response, confirming exposure.

Cytokine analysis conducted on Day 30 (1 day after the third dose) and Day 58 (1 day after the fifth and final dose) revealed higher IFN-γ, IL-6, and KC/GRO activity, with higher levels on Day 30 than on Day 58. There were also increases in IL-10 and TNF-α activity on these days, with higher levels on Day 58 than on Day 30. After a 2-week recovery period, all cytokine levels had returned to baseline. Higher levels of ADA were also apparent on Days 30 and 58 and were still present at the end of the recovery period. On Days 30 and 58, ELISpot investigations revealed an antigen-specific IFN-γ response, which was still present as the end of the 14-day recovery period.

Microscopic examination found minimal or slight inflammatory cell infiltrate at the injection sites and minimal to moderate increased lymphocyte cellularity and minimal or slight increased macrophage cellularity in the axillary lymph nodes. These findings were consistent with a normal immunological response to a foreign antigen and with the expected pharmacological response and not considered adverse.

Minimal to moderate increased extramedullary haematopoiesis correlating with macroscopic enlargement and higher weights in the spleen and a minimal to slight increase in cellularity of the bone marrow were observed. The increased cellularity of the bone marrow is likely related to a normal immunological response to a vaccine where priority is given to some white blood cell lineages at the expense of red blood cell and platelet precursors. In rodents, it manifested as extramedullary haematopoiesis, since this is typically met by an adaptive response in the spleen, with production of red blood cells. This is also consistent with the lower red blood cell and platelet counts seen in the peripheral blood. The general reduction in white blood cell lineages seen in the peripheral blood may be related to their margination into tissues such as the lymph nodes and dosing sites to accommodate an active immune response. All these changes were mild, showed good evidence of reversibility, and were not considered adverse. The higher plasma globulin concentrations observed are likely to reflect the increased production of immunoglobulin consistent with the ADA response and possibly also the presence of acute phase proteins. The changes in the peripheral blood were generally

more marked in females and consistent with the greater INF-y response seen in the ELISpot assay.

Slightly lower alanine aminotransferase, glucose, cholesterol, and triglyceride were also observed. This can reflect minor liver damage; however, there were no histological changes observed in the liver of treated animals, and these are more likely to reflect minor normal fluctuations in food intake or metabolism. Therefore, these differences were considered to be fortuitous and unrelated to treatment.

As noted above (Section 1.2), due an unforeseen supply issue with 3D (6-acyl) MPLA, Montanide is to be used as the adjuvant in this clinical study. In a repeat dose toxicology study where Montanide was administered intramuscularly up to 430 mg/mL, there was no mortality, but nodules did develop at the injection site. There were increases in the weight of popliteal lymph nodes in male and female animals and decrease in spleen weight in male animals. In other toxicology studies, there were no identified effects on embryofoetal or reproductive function and Montanide was not genotoxic (Montanide IB). Although toxicology studies were performed via intramuscular route of administration, over 300 clinical studies have been conducted with Montanide, the majority of which have been conducted with SC administration supporting the use of Montanide via the SC route (Montanide IB).

1.4. Summary of Nonclinical Pharmacokinetics

Not applicable.

1.5. Summary of Clinical Experience

There is no prior human experience with OVM-200.

1.6. Study Rationale

As outlined in Section 1.1 and Section 1.2, the rationale for using OVM-200 as a therapeutic cancer vaccine in patients with locally advanced or metastatic NSCLC, ovarian cancer, and prostate cancer is based on the suggestion that is ROPs could be more effective than the native protein in antigen presentation and the positive results in mice. The 3 disease types were selected for investigation as all 3 having been found with high occurrence of survivin overexpression (see Section 3.3 for full details of patient population).

This is the first time OVM-200 will be administered to humans. An objective of Phase 1a of this study is to determine the dose of OVM-200 for use in Phase 1b of the study. This information, together with the safety, tolerability, and efficacy data, will help establish the doses and dosing regimen for future studies.

1.7. Benefit-risk Assessment

This study will represent the first time that the vaccine OVM-200 has been administered to humans.

A program of nonclinical studies, including toxicology studies, has been performed in relevant animal species, as mentioned in the above sections. Pharmacology studies support

the potential for OVM-200 to generate an immune response to survivin and to consequently slow tumour growth. In the toxicology study, after 8 weeks of SC injection treatment with OVM-200 in combination with the commercially available adjuvant 3D (6-acyl) MPLA in mice, the treatment was considered well tolerated. Some inflammatory changes at the SC injection sites were noted. Treatment-related findings with regard to haematology and blood chemistry investigations and findings at macro and microscopic investigations in the spleen, axillary lymph nodes, and injection sites were considered to reflect expected immunologically mediated change and not considered adverse effects.

1.7.1. Potential Risks

The risks of participation are primarily those associated with adverse reactions to the investigational medicinal product (IMP), principally immune-mediated reactions including potentially anaphylactic reactions and local site reaction, although there may also be some discomfort from collection of blood samples and other study procedures.

The incidence of anaphylactic reactions or other serious hypersensitivity reactions with vaccines is generally very rare (9). Postvaccination anaphylaxis in the United States has a reported rate of 1.3 cases per 1 million doses administered (10). In an Australian long-term surveillance study following human papilloma virus vaccine, the rate of anaphylaxis was 0.32 per 100 000 doses, which the authors considered consistent with published rates; urticaria was seen in approximately 2.5% of people and rash in 2.4% to 3% (11). Nevertheless, cases of anaphylaxis have been reported in Phase 1 studies of potential anticancer vaccines. One out of 45 patients dosed with a novel multi-peptide therapeutic vaccine (11 injections over 24 weeks) experienced anaphylaxis (12). In another study, 1 out of 33 patients dosed with folate immune therapy (EC90 vaccine administered with GPI-0100 adjuvant) experienced anaphylaxis (13). Investigators and staff will have treatments for anaphylaxis readily available at the time of OVM-200 dosing. The majority of anaphylactic reactions to vaccines occur within 2 hours of treatment and therefore patients will be required to stay at the clinical study site for a minimum of 4 hours following injection of OVM-200 in Phase 1a and 2 hours in Phase 1b (10, 14).

The Montanide adjuvant has been associated with injection site reactions (including injection site pain, oedema and granuloma); myalgia; headache; gastro-intestinal disorders; fatigue and fever, with adverse effects being mainly mild or moderate in intensity (8).

1.7.2. Potential Benefits

The goal of treatment with the Investigational Medicinal Product (IMP) will be to induce a reduction in tumour size and/or to delay progression of the cancer; however, it should be assumed that most if not all study participants in this initial clinical study will experience no detectable benefit in terms of cancer control.

1.7.3. Overall Risk-benefit Assessment

The study will enrol patients with locally advanced or advanced cancer that have already received at least 1 line of cancer therapy (or in the case of prostate cancer are currently stable on an antihormonal treatment) and are currently not considered to require further therapy during the primary treatment and follow-up phases of this study. Patients will be stable without evidence of progressive disease after completion of a prior therapy course and hence

do not require any established therapy for their cancer at this stage of their disease, or they have exhausted currently recognised treatment options. For example, patients with locally advanced or advanced ovarian cancer may undergo initial treatment with surgery followed by a course of 6 cycles of platinum-based chemotherapies and then observed (with imaging, cancer antigen-125 (CA-125) and clinical observation) before consideration of therapy. Similarly, NSCLC patients may complete a number of cycles of therapy before further therapy is considered. Prostate cancer patients typically have extended periods of disease stability of several months or even years while on androgen deprivation therapies; which can be continued in this study.

In addition, the eligibility and stopping criteria ensure that patients are not being deprived of established efficacious cancer therapy prior to study entry and during the study. For example, a patient will be withdrawn from the study if they demonstrate a clinically relevant sign or symptom or clinical deterioration/progression that, in the opinion of the investigator (or designee), warrants patient withdrawal from treatment. Thus, all patients will receive standard of care prior to enrolling in this study and will not be deprived of any recommended treatment options that are known to have curative or life-prolonging potential.

Although generally only patients who had exhausted all other options would be enrolled in a First-in-human (FIH) study, these patients may be immunocompromised. Therefore, the opportunity to generate and measure an immune response may be limited. The potential to include patients earlier in the treatment continuum, but without preventing them from receiving treatment with other therapies, therefore represents the best potential opportunity to generate and measure immune response to OVM-200.

Given the anticipated safety profile and the available toxicology data, the risk to the patient is considered relatively low but will be appropriately mitigated through inclusion/exclusion criteria and stopping rules. Given the mode of action of OVM-200, the risk of harmful pharmacokinetic or pharmacodynamic interactions with current or subsequent cancer therapies is considered to be low. Based on the nonclinical data available to date, the conduct of the study is considered justifiable using the selected dose levels and dosage regimen of OVM-200 as specified in this protocol. Given the available pharmacological data and experience with other survivin vaccines, the selected dose offers a reasonable potential to provide benefit to patients. However, there is also a conservative calculation of a safe starting dose (> 1000-fold less than a tolerated dose in mice, on a body weight-adjusted basis) and sequential inclusion of patients in the study to limit exposure (See Section 3.4). In addition, there is close monitoring of safety by clinicians in clinical units with expertise in conducting Phase 1 clinical studies. Patients will dose in small cohorts of 3 patients with initial single-patient sentinel dosing at each dose level studied (see Section 3.5). Patients will be observed for at least 4 hours post dose in Phase 1a and 2 hours in Phase 1b and, if necessary, treated for any allergic or other reactions. The majority of anaphylactic reactions to vaccines occur within 2 hours of treatment and therefore an observation period of 2 to 4 hours is considered acceptable to mitigate this risk (10, 14).

A SRC will meet regularly to review emerging safety data within the study. Clear stopping rules are defined (Section 3.7) and the study will be discontinued in the event of any new findings that indicate a relevant deterioration of the risk-benefit relationship that would render continuation of the study unjustifiable.

As this is a FIH study with an experimental agent, it should be assumed that most if not all study participants will experience no benefit in terms of cancer control, and the primary benefit from this study is to support later stage development work. More information about the known and expected benefits, risks, and reasonably anticipated adverse events (AEs) can be found in the separate OVM-200 IB.

2. OBJECTIVES AND ENDPOINTS

2.1. Objectives

The primary objective of the study is:

- To characterise the safety and tolerability of OVM-200 in patients with tumour types known to over-express survivin (non-small cell lung cancer [NSCLC], ovarian cancer, and prostate cancer).

The secondary objectives of the study are:

- To determine the immune response following administration of OVM-200 in patients with tumour types known to over-express survivin
- To determine the recommended dose of OVM-200 for further development in patients with tumour types known to over-express survivin.

The exploratory objectives of the study are:

- To conduct a preliminary exploration of the antitumour activity of OVM-200 in patients with tumour types known to over-express survivin
- To evaluate the correlation of survivin expression with response to the administration of OVM-200.

2.2. Endpoints

2.2.1. Safety Endpoint

The primary endpoint (safety and tolerability) for this study is the occurrence and intensity of AEs.

2.2.2. Efficacy Endpoints

The secondary endpoint (efficacy) is the immune response to OVM-200 as measured by ELISpot for T-cell responses and ELISA for antibody responses.

The exploratory efficacy endpoints are as follows:

- Disease progression and tumour response evaluated using Response Evaluation Criteria in Solid Tumour (RECIST) v1.1 including objective response rate (ORR) over 24 weeks, time to response (TTR), duration of response (DoR), progression-free survival (PFS), and OS, as appropriate. Bone lesions in prostate

cancer patients will be evaluated using the methodologies described by the Prostate Cancer Working Group 3 (PCWG3) (15).

- In ovarian cancer patients, tumour marker CA-125 measured and evaluated according to Gynecologic Cancer Intergroup Criteria (GCIC) (16).
- In prostate cancer patients, tumour markers prostate-specific antigen (PSA) and total alkaline phosphatase (ALP) as per PCWG3 response criteria (15).
- Survivin expression will be correlated to response (based on immune response, tumour assessments, and tumour marker measurements) achieved after OVM-200 administration.

3. INVESTIGATIONAL PLAN

This will be a multicentre, open-label, nonrandomised, FIH, Phase 1a/1b study of OVM-200 in patients with locally advanced or metastatic NSCLC, ovarian cancer, and prostate cancer.

3.1. Overall Study Design and Plan

This is a 2-part study in patients with:

i. NSCLC
ii. ovarian cancer, and
iii. prostate cancer.

The first part (Phase 1a) comprises a FIH multiple-dose, sequential-cohort design 3+3 to establish a dose of OVM-200 that is safe and tolerable, and that elicits an immune response in humans.

This dose will be taken forward into the second part (Phase 1b) of the study. Phase 1b will further assess the safety and tolerability of the selected dose and investigate the immune and tumour response in 3 expansion cohorts of additional patients with NSCLC, ovarian cancer, and prostate cancer.

A schedule of assessments is presented in Appendix 6.

An overview of the study design for both phases of the study is shown in Figure 2.

Figure 2: Study Schematic

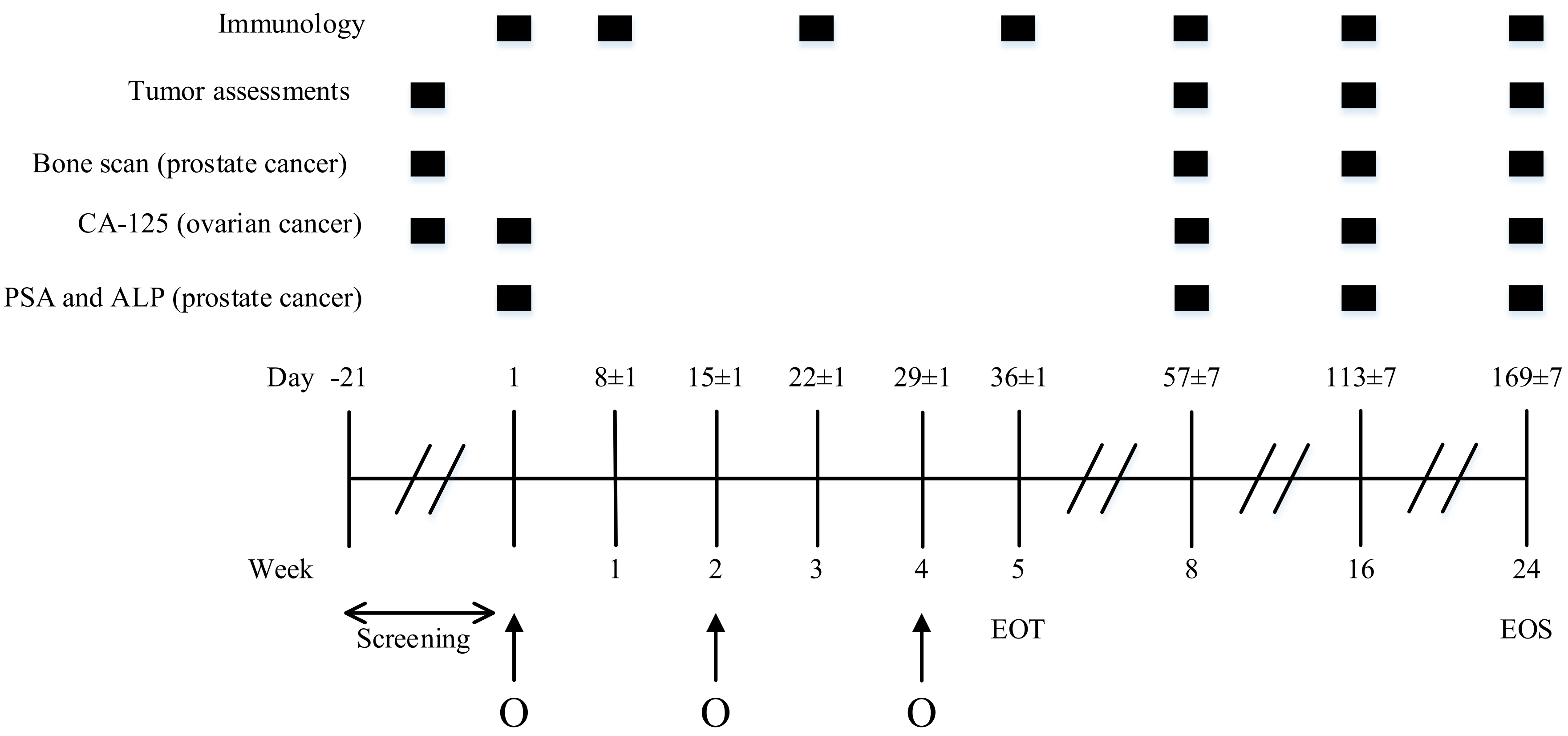

Abbreviations: ALP = total alkaline phosphatase; CA-125 = cancer antigen-125; EOS = end of study; EOT = end of treatment; NSCLC = non-small cell lung cancer; O = OVM-200 dose; PSA = prostate-specific antigen.
Note: Immunology sampling on Weeks 1 and 3 for Phase 1a only.

3.1.1. Phase 1a

Phase 1a will comprise a multiple-dose, sequential-cohort, 3+3, design to establish the dose of OVM-200 that is safe and tolerable and that elicits an immune response in patients with NSCLC, ovarian cancer, or prostate cancer. Overall, 3 to 24 patients will be studied in 1 to 4 dose levels (Cohorts A1 to A4), with each cohort consisting of 3 to 6 patients.

OVM-200 will be administered after at least 1 line of systemic cancer treatment is completed with the exception of supportive therapies and androgen deprivation therapies for prostate cancer patients, which may be continued. After a screening period of up to 21 days, eligible patients will arrive at the site on the first day of dosing (Day 1) to confirm eligibility, complete predose baseline assessments, and receive the first dose of OVM-200. Patients will receive 3 doses of OVM-200 at 2-week intervals. The patients will remain at the site for at least 4 hours after each dose for observation. Between dose administrations, patients will return for weekly visits through the end-of-treatment (EOT) visit on Week 5. There will be additional visits on Weeks 8 and 16 and an end-of-study (EOS) visit on Week 24.

A SRC will review all available cumulative safety, tolerability, and immune response data throughout the trial. Any notable findings following treatment will be communicated to all sites immediately. Phase 1a will be a standard 3+3 design with up to 4 cohorts with dose escalation as described in Section 3.5 and stopping rules as defined in Section 3.7. In each cohort, 1 sentinel patient will be dosed initially with 2 additional patients dosed at the same dose level at least 2 weeks later. Cohort 4 will not proceed if a clear plateau in immune response (defined as the absence of a significant difference in ELISpot response between doses using geometric means of at least 2 standard deviations) is observed in the previous cohorts.

Following review of all available cumulative safety, tolerability, and immune response data through Week 5 (1 week after the final vaccination) from all cohorts, the SRC will recommend a dose to be carried forward into Phase 1b in accordance with Section 3.1.2.

The planned dose levels for Phase 1a are shown in Figure 3.

Figure 3: Planned Dose Levels (Phase 1a)

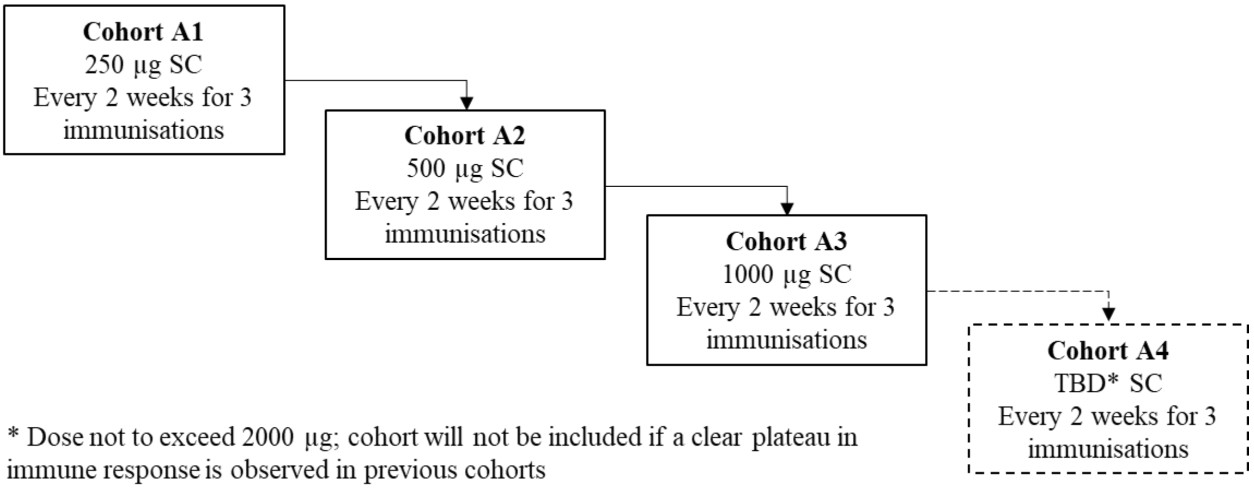

* Dose not to exceed 2000 µg; cohort will not be included if a clear plateau in immune response is observed in previous cohorts

Abbreviations: SC = subcutaneous; TBD = to be determined

The total duration of study participation for each patient (from screening through EOS visit) is anticipated to be up to approximately 190 days.

3.1.2. Criteria for Transition to Phase 1b

Transition to Phase 1b will occur only if the SRC establish that there is a dose identified that:

1. Has an acceptable safety profile defined as 1 or fewer Dose-limiting toxicity (DLT)s within a cohort and no other safety findings that would, in the opinion of the SRC prevent dosing in Phase 1b, and
2. Results in an immune response defined as a difference between preimmunisation and postimmunisation geometric means in ELISpot response of 2 standard deviations or more across three patients.

The decision will be taken after review of all available data when all Phase 1a patients have, as a minimum, completed their DLT assessment follow up period, ie 1 week of follow up after the final OVM-200 injection, and the Week 5/EOT immune response data are available for all Phase 1a patients. This time point should represent the maximum T-cell response following treatment with OVM-200. The definition of immune response to OVM-200 allows for variation in the baseline immune response of an individual patient but represents a significant change from baseline.

3.1.3. Phase 1b

Phase 1b will comprise a multiple-dose, multiple-cohort study using the established dose from Phase 1a to confirm safety and tolerability and to investigate the immune and tumour response in 3 expansion cohorts of at least 8 patients with i) NSCLC, ii) ovarian cancer, and iii) prostate cancer. Overall, a minimum of 24 patients will be studied in 3 therapeutic indications (Cohorts B1 to B3), with each cohort consisting of approximately 8 patients.

More than 8 patients may be enrolled in a cohort, for a maximum of 28 patients total in this Phase of the study. If more than 8 patients are enrolled in 1 of the cohorts (up to a maximum of 10 patients), fewer patients may be enrolled in either or both of the other cohorts so that there is a total of 24 to 28 patients from all 3 cohorts in this phase.

OVM-200 will be administered after at least 1 line of systemic cancer treatment is completed, with the exception of supportive therapies and androgen deprivation therapies for prostate cancer patients, which may be continued. After a screening period of up to 21 days, eligible patients will arrive at the site on the first day of dosing (Day 1) to confirm eligibility, complete predose baseline assessments, and receive the first dose of OVM-200. Patients will receive 3 doses of OVM-200 at 2-week intervals. The patients will remain at the site for at least 2 hours after each dose for observation. Between dose administrations, patients will return for weekly visits through the EOT visit on Week 5. There will be additional visits on Weeks 8 and 16 and an EOS visit on Week 24.

The total duration of study participation for each patient (from screening through EOS visit) is anticipated to be up to approximately 190 days.

3.2. Study Start and End of Study Definitions

The start of the study is defined as the date the first patient in the study signs an informed consent form (ICF). The point of enrolment occurs at the time of patient number allocation.

The end of the study is defined as the date of the last assessment for the last patient in the study (scheduled or unscheduled).

3.3. Discussion of Study Design

For Phase 1a, a sequential-cohort, ascending-dose, 3+3 design has been chosen for safety reasons. This is because OVM-200 is in the early stages of clinical development, with Phase 1a of the study being the first time it will be administered to humans. Based upon the nonclinical data, the duration of the treatment period is considered adequate to achieve the study objectives. The treatment period directly relates to the proposed protocol following the outcomes of the dose escalation study, being 3 immunisations 2 weeks apart with a follow-up period, post-treatment, up to a maximum of 24 weeks after the start of the study. The 24-week follow-up period is considered a reasonable maximum period for assessments of safety and efficacy in a Phase 1 study of an advanced cancer population with a primary goal of exploring short-term safety and tolerability.

SC doses have been chosen for both parts of the study, as this is the intended clinical route of administration. For safety reasons, sentinel dosing will be used in Phase 1a, such that 1 patient will be dosed at least 2 weeks before the remaining 2 patients. A 3+3 design is commonly employed for Phase 1 oncology studies as it minimises the number of patients given an ineffective anticancer agent as well as those given a drug with unacceptable toxicity (17). Sentinel dosing is not considered necessary in Phase 1b as exposure is not predicted to exceed an exposure previously shown to be safe and tolerated in Phase 1a. Details of the dosing regimen used for Phase 1b of the study will be documented in the TMF (Trial Master File).

For this Phase 1 safety study, 3 disease types were selected for investigation, all 3 having been found with high occurrence of survivin overexpression. Patient populations were chosen based on the following:

- The likelihood of tumours being positive for survivin expression (Table 1).
- Sufficient patients in late-stage disease progression where treatment options have effectively been exhausted or will not be enacted within 9 weeks of starting the study.
- The ability to monitor efficacy via measurement of blood markers.

Table 1: Expression of Survivin Protein

Cancer Type	Percentage Expression
Lung	85.5%
Ovarian	73.5%
Prostate	99%

Source: (18)

Enrolment will be irrespective of survivin status; however, pretreatment samples must be available for survivin expression assay and an exploratory analysis of relationships between survivin expression and efficacy endpoints will be performed.

Immune response will be measured by ELISpot and ELISA to provide scientific proof-of-concept. Traditional markers of immunogenicity are serum antibody titres, measured by ELISA, and CD8+ T-cells, assessed by an interferon gamma release assay/ELISpot. This Phase 1 study is measuring the primary antibody response to immunisation with the survivin ROP and the generation of cytotoxic T-cells targeting survivin peptides. In Phase 1a, samples will be taken 1 week after vaccinations, in conjunction with the postdose safety evaluation visits for the patients' convenience, to allow the maximum response. The final sample is taken 1 week after the last vaccination, in line with preclinical studies.

Other assessments are detailed in Section 7 and Appendix 3.

3.3.1. Dose Interval

Three to 6 patients will be dosed at each dose level in Phase 1a. At each dose level, 1 sentinel patient will be dosed initially with 2 additional patients dosed at the same dose level at least 2 weeks later if no dose-limiting toxicities (DLTs) or other safety and tolerability concerns are observed in the sentinel patient (see Section 3.6 for DLT definition). If a DLT or other notable safety event is experienced in the sentinel patient, the SRC will review all available data before deciding on the continuation of dosing in subsequent patients (see Section 3.7). Patients will receive each dose 2 weeks apart as was performed in the supporting toxicology study with OVM-200 and in multiple studies with the adjuvant Montanide.

3.4. Selection of Doses in the Study

A starting dose of 250 µg has been selected for this FIH study. This is based on the doses studied in non-clinical studies and existing experience with similar products in clinical

studies. The highest dose studied in toxicology and pharmacology studies in mice was 200 µg which was well tolerated and generated an immune response accompanied by evidence of anti-tumour effects. Although not necessarily appropriate for a therapeutic vaccine such as OVM-200, based on body weight adjustment a dose of 250 µg represents a 2400-fold safety margin from the toxicology study. Earlier studies using a survivin-targeting peptide cancer vaccines used 250 or 500 µg of peptide vaccines (19). Another survivin vaccine study used 500 µg (20). Finally, other cancer vaccine studies have started with doses of 100 or 500 µg (21-24).

Initial studies with ROP vaccines in mouse models utilised 3 immunisations at 3-week intervals to provide prophylactic protection against growth of post immunisation inoculated melanoma cells. Later investigations in mouse models showing antitumour activity for drug candidates in therapeutic programs with immunisations after the introduction of tumour cells have been run with 3 immunisations at 1- and 2-week intervals. The Good Laboratory Practice (GLP) toxicology study in mice was run at 2-week intervals. Therefore, it was decided for this FIH study to space the immunisations at 2-week intervals to match the toxicology study.

The peak T-cell response following an immunisation is commonly reported as occurring between 7 and 10 days, with the formation of memory cells on a longer time course (13, 25). This peak roughly corresponds to the eradication of a pathogen. Over the next 7-10 days 90% to 95% of antigen-specific T-cells then die off, leaving behind a pool of memory cells (13, 25). This peak period can be extended with slower release of antigen, such as from an SC depot, supporting the extension of the interval for repeat immunisations. There are numerous examples of Phase 1 clinical studies for peptide cancer vaccines that are commonly spaced at 1-, 2-, and 3-week intervals (26-29). This also supports the 2-week interval in this study.

To limit the number of patients in the dose escalation phase who might receive an ineffective dose, a doubling increase rate from 250 µg is proposed, which has a reasonable chance to produce an optimum dose. Because it is unlikely that a DLT will be reached as it would with a cytotoxic drug, the greater need is to avoid underdosing. Doubling allows the study dose to approach a maximal response with greater precision at the lower end of the dose range. Other studies scaled from 100 µg to 1000 µg and 500 µg to 1500 µg (21, 23, 24). The dose for cohort 4 will be determined by the SRC following review of the available data from cohorts 1-3 and will not exceed a maximum dose of 2000 µg which represents the highest injectable volume in an individual patient of 2 mL. This dose will still represent a 300-fold safety margin compared to the dose studied in the toxicology study. Cohort 4 will not be conducted if a clear plateau in immune response is demonstrated in the previous 3 cohorts.

A maximum dose of 1 mL of Montanide, will be used in this clinical study which is within the range used extensively in clinical trials with Montanide to date (Montanide IB).

Details of all doses administered in Phases 1a and 1b of the study will be documented in the TMF.

3.5. Dose Escalation

All dose escalations decisions will be made by the SRC.

Three patients will initially be dosed in a cohort. If no DLT is observed in any of these patients to week 5 (1 week after the final vaccination in the last patient to be dosed) dosing will commence in the next cohort. If one patient develops a DLT at a specific dose, an additional three patients will be enrolled into that same dose cohort (note: patient screening activities for subsequent cohorts may commence after the final patient in a cohort has had their Week 3 follow up visit and there is no DLT observed in any patient in that cohort). Development of DLTs in more than 1 of 6 patients in a specific dose cohort suggests that the MTD has been exceeded, and further dose escalation will not be pursued. In addition, cohort 4 will not proceed if a clear plateau in immune response is observed in the previous 3 cohorts defined as the absence of a significant difference in ELISpot response between doses using geometric means of at least 2 standard deviations). The dose in cohort 4 will be determined by the SRC based on all available safety and immunogenicity data to week 5 (1 week after the final vaccination in the last patient to be dosed) and will not exceed 2000 µg.

The SRC will convene as soon as possible after a DLT is reported to review data and determine the course of action for any further patients planned to receive the same dose. The SRC can recommend that additional cohorts of patients are studied at the same or lower doses in subsequent cohorts.

Table 2: Proposed Investigational Medicinal Product Dose Levels for Phase 1a

Dose Level	Dose of each Vaccination (µg)
1	250
2	500
3	1000
4	To be determined up to a maximum of 2000

The dose in Phase 1b will not exceed the dose safely administered in Phase 1a. The SRC will review data from Phase 1a and make a recommendation on the Phase 1b dose as per Section 3.1.2.

The SRC will continue to review accumulating data in Phase 1b and make recommendations regarding dosing and safety monitoring.

3.6. Dose-limiting Toxicities

A DLT is defined as any of the following occurring from first dose to week 5 (1 week following the final dose of OVM-200) and assessed as at least possibly related to study drug by the investigator:

- Immune-mediated toxicity or allergic reaction grade ≥ 3
- Anaphylaxis or cytokine release syndrome (any grade)
- Nonhaematological AE grade ≥ 3 with the exception of alopecia and fatigue
- Neutropenia grade ≥ 3 that does not resolve to grade ≤ 1 within 3 days despite best supportive care
- Febrile neutropenia grade ≥ 3
- Neutropenia grade ≥ 4

- Thrombocytopenia grade ≥ 3 that does not resolve to grade ≤ 1 within 3 days despite best supportive care
- Thrombocytopenia grade ≥ 4

3.7. Stopping rules

The stopping rules within the sections below will apply.

3.7.1. Individual patient (Phase 1a and 1b)

Dosing of an individual patient will stop immediately if any of the criteria outlined in Section 4.4 are met.

3.7.2. Phase 1a

3.7.2.1. Within a cohort

Dosing will be paused within a cohort in the event of a DLT (Section 3.6) or other safety concern that, in the opinion of the SRC, represents a risk to other patients within the cohort. Dosing may restart following detailed review of the available data upon the recommendation of the SRC. Dosing will be stopped if two or more patients within a cohort experience a DLT.

3.7.2.2. Between Cohorts

In accordance with Section 3.5, dose escalation will be stopped if 2 or more patients experience a DLT or other safety concern that is considered by the SRC to represent a risk to further dose escalation.

3.7.3. Progression to Phase 1b

Progression to Phase 1b will be halted if it has not been possible for the SRC to identify an appropriate dose for use in Phase 1b as defined in Section 3.1.2.

3.7.4. Phase 1b

In the event of 2 or more patients experiencing a DLT or the SRC makes a recommendation based on other important, safety concerns, dosing in Phase 1b of the study will be paused. Dosing may restart following detailed review of the available data only upon the recommendation of the SRC.

3.7.5. Termination of the study

The study will be stopped if any of the criteria listed in Section 4.5 are met.

4. SELECTION OF STUDY POPULATION

4.1. Inclusion Criteria

Patients must satisfy all of the following criteria at the screening visit unless otherwise indicated:

1. Histologically confirmed metastatic or locally advanced inoperable NSCLC, ovarian cancer, or prostate cancer that have already received at least 1 line of approved cancer therapy and either: exhausted current recognized treatment options; or are stable in a planned treatment-free interval following completion of a set course of treatment; or in the case of prostate cancer, are currently stable on an antihormonal treatment.
2. Are not receiving active cancer treatment other than supportive therapies or androgen deprivation therapies for prostate cancer, which may be continued, and, in the opinion of the investigator, are not anticipated to require further approved cancer treatment options until the Week 8 assessment (up to 9 weeks) after the first dose of OVM-200 per standard of care.
3. At least 1 measurable lesion that can be accurately assessed at baseline by computed tomography (CT)/magnetic resonance imaging (MRI) and is suitable for repeated assessment (NSCLC only).
4. Age ≥ 18 years and ≤ 75 years.
5. Eastern Cooperative Oncology Group (ECOG) performance status ≤ 2 (Section 7.2.6).
6. Predicted life expectancy ≥ 3 months.
7. Adequate bone marrow, renal, and hepatic function, with:
   - Haemoglobin ≥ 9 g/dL (can be post-transfusion
   - Absolute neutrophil count (ANC) ≥ 1.0 × 10^6 cells/L.
   - Platelets ≥ 100 × 10^9 cells/L (cannot be post-transfusion).
   - Aspartate aminotransferase (ALT) and alanine aminotransferase (AST) ≤ 3 × upper limit of normal (ULN).
   - Albumin ≥ 3 g/dL.
   - Urine dipstick for proteinuria is negative or trace. Patients may be included with results of 1+ if they have a spot urinary albumin creatinine ratio of either (i) ≤ 3 mg/mmol or (ii) > 3 mg/mmol and < 70 mg/mmol with a 24-hour urinary protein < 0.2 g/24 hours.
8. All toxicities attributed to prior anticancer therapy other than alopecia or those attributed to antihormonal therapies for prostate cancer have resolved to grade 1 or their pretherapy level before the first dose of study drug. Patients with toxicities (other than renal toxicities) attributed to prior anticancer therapy that are not expected to resolve and result in long-lasting sequelae, such as neuropathy after platinum-based therapy, or those attributed to androgen deprivation therapies for prostate cancer are permitted to enrol if the condition is stable and considered unlikely to affect interpretation of study data.
9. Females must not be pregnant or lactating, and females of childbearing potential and males must agree to use contraception as detailed in Appendix 4.
10. Able to comprehend and willing to sign an ICF and to abide by the study restrictions.

4.2. Exclusion Criteria

Patients will be excluded from the study if they satisfy any of the following criteria at the screening visit unless otherwise stated:

1. Known history or evidence of significant immunodeficiency due to underlying illness. Patients with a condition requiring systemic treatment with either corticosteroids (> 10 mg daily prednisolone equivalent) or other immunosuppressive medications within 14 days of the first dose of study drug. Inhaled or topical steroids and adrenal replacement steroids are permitted in the absence of autoimmune disease.
2. Patients with a history of or active, known, or suspected autoimmune disease or a syndrome that requires systemic or immunosuppressive agents. Patients with vitiligo, type I diabetes mellitus, residual hypothyroidism due to autoimmune disease only requiring hormone replacement, psoriasis not requiring systemic treatment, or conditions not expected to recur in the absence of an external trigger are permitted to enrol.
3. Prior therapy with an anticancer vaccine; anti-PD-1, anti-PD-L1, anti-PD-L2, anti-CD137, or anti-CTLA-4 antibody; or any other antibody or drug specifically targeting T-cell co-stimulation or immune checkpoint pathways in the 28 days before the first dose of study drug.
4. Administration of an investigational drug in the 28 days or 6 half-lives (whichever is longer) before the first dose of study drug.
5. Major surgery or treatment with any chemotherapy, or radiation therapy for cancer in the 28 days before the first dose of study drug.
6. Active infection requiring antibiotics or physician monitoring, or recurrent fevers (> 38.0°C) associated with a clinical diagnosis of active infection.
7. Active viral disease, positive test for hepatitis B virus using hepatitis B surface antigen test, or positive test for hepatitis C virus (HCV) using HCV ribonucleic acid or HCV antibody test indicating acute or chronic infection. Positive test for human immunodeficiency virus (HIV) or acquired immunodeficiency syndrome; testing is not required in the absence of history.
8. Receipt of any vaccine within 28 days before the first dose of study drug.
9. Other prior malignancy within the previous 3 years, except for local or organ-confined early stage cancer that has been definitively treated with curative intent and does not require ongoing treatment, has no evidence of residual disease, and has a negligible risk of recurrence and is therefore unlikely to interfere with the primary and secondary endpoints of the study, including response rate and safety and tolerability.
10. Symptomatic brain metastases or any leptomeningeal metastasis.
11. Any serious or uncontrolled medical disorder (including cardiovascular, respiratory, renal, or autoimmune disease) that, in the opinion of the investigator or the medical monitor, may increase the risk associated with study participation or study drug administration, impair the ability of the patient to receive protocol therapy, or interfere with the interpretation of study results.

12. History of allergic reaction or hypersensitivity to any component of the OVM-200 therapeutic vaccine or adjuvant.

4.3. Patient Number and Identification

Screen failures are defined as patients who consent to participate in the clinical study but are not subsequently included in the analysis population due to failure of the eligibility criteria. A minimal set of screen failure information is required to ensure transparent reporting of screen failure patients to meet the Consolidated Standards of Reporting Trials publishing requirements and to respond to queries from regulatory authorities. Minimal information includes demography, screen failure details, eligibility criteria, and any Serious Adverse Events (SAE)s.

Screen failures will be replaced.

Patient numbering and enrolment will be detailed in a separate enrolment plan.

4.4. Patient Withdrawal and Replacement

A patient is free to withdraw from study treatment at any time. In addition, a patient will be withdrawn from study treatment if any of the following criteria are met:

- Change in compliance with any inclusion/exclusion criterion that is clinically relevant and affects patient safety as determined by the Investigator (or designee)
- Noncompliance with the study restrictions that might affect patient safety or study assessments/objectives, as considered applicable by the investigator (or designee)
- Occurrence of a DLT (Section 3.6)
- Any clinically relevant sign or symptom or clinical deterioration/progression that, in the opinion of the investigator (or designee), warrants patient withdrawal from treatment
- Other (e.g., start of new therapy or discretion of the investigator [or designee]).

A patient is free to withdraw from the study as a whole at any time. In addition, a patient will be withdrawn from the study if any of the following criteria are met:

- Death
- Lost to follow-up
- Sponsor's decision to terminate the study.

If a patient is withdrawn from either treatment or the study, the sponsor will be notified and the date and reason(s) for the withdrawal will be documented in the patient's electronic case report form (eCRF). Patients who are withdrawn from study treatment will continue to attend the scheduled study visits. If a patient is withdrawn from the study, efforts will be made to perform all EOT/end-of-study assessments, if possible.

If a patient is withdrawn from treatment or the study for reasons other than consent withdrawal, other procedures may be performed at the investigator's (or designee's) and/or

sponsor's discretion. If the patient is in-house, these procedures should be performed before the patient is discharged from the site. The investigator (or designee) may also request that the patient return for an additional follow-up visit. All patients who are withdrawn from study treatment will be followed until resolution of all their AEs or until the unresolved AEs are judged by the investigator (or designee) to have stabilised.

Patients who are withdrawn for reasons not related to study drug may be replaced following discussion between the investigator and the sponsor. Patients withdrawn as a result of AEs thought to be related to the study drug will not be replaced.

4.5. Study Termination

The sponsor reserves the right to close a study site or terminate the study at any time for any reason at the sole discretion of the sponsor. Study sites will be closed upon study completion. A study site is considered closed when all required documents and study supplies have been collected and a study site closure visit has been performed.

The investigator may initiate study site closure at any time, provided there is reasonable cause and sufficient notice is given in advance of the intended termination. Reasons for the early closure of a study site by the sponsor or investigator may include, but are not limited to:

- Failure of the investigator to comply with the protocol, the requirements of the ethics committee (EC) or local health authorities, the sponsor's procedures, or Good Clinical Practice (GCP) guidelines
- Inadequate recruitment of participants by the investigator.

Reasons for study termination may include, but are not limited to:

- SRC recommendation based on review of accumulating data
- AEs unknown to date (ie, not previously reported in any similar investigational study drug study with respect to their nature, severity, and/or duration)
- Increased frequency and/or severity and/or duration of known, anticipated, or previously reported AEs (this may also apply to AEs defined on Day 1 as baseline signs and symptoms)
- Medical or ethical reasons affecting the continued performance of the study
- Difficulties in the recruitment of patients
- Cancellation of drug development.

5. STUDY TREATMENTS

5.1. Description, Storage, Packaging, and Labelling

The IMP (2 mg/mL OVM-200 solution and Montanide ISA 51 VG adjuvant) will be supplied by the sponsor (or designee), along with the batch/lot numbers and certificates of analysis. The IMP will be provided in vials and stored according to the instructions on the label.

All IMPs will be stored at the study site in a location that is locked with restricted access.

The bulk drug container and unit dose containers will be labelled in accordance with national laws and regulations (30). The individual unit doses will be prepared by qualified site staff.

5.2. Study Treatment Administration

OVM-200 will be combined with an equal volume of Montanide to form an emulsion following the pharmacy manual (available separately). Doses from this preparation will be injected in the upper arm, or elsewhere if necessary, SC. Detailed information describing the preparation and administration will be provided in the pharmacy manual.

5.2.1. Dose Modification

Dose reductions will not be allowed. If a dose reduction is needed, the patient should be discontinued from treatment and all EOT assessments completed.

If a patient is not able to receive study drug at the planned timepoint, dosing can be delayed up to 1 week with medical monitor approval. If doses cannot be given within 1 week or if a dose is omitted, the patient will be discontinued from treatment and all EOT assessments completed.

5.3. Randomisation

Not applicable.

5.4. Blinding

This is an open-label study and will not be blinded.

5.5. Treatment Compliance

The following measures will be employed to ensure treatment compliance:

- All doses will be administered under the supervision of suitably qualified study site staff.
- At each dose preparation occasion, a predose and postdose inventory of IMP will be performed.

5.6. Drug Accountability

The investigator (or designee) will maintain an accurate record of the receipt of the study supplies received. In addition, an accurate drug disposition record will be kept, specifying the amount dispensed to each patient and the date of dispensing. This drug accountability record will be available for inspection at any time. At the completion of the study, the original drug accountability record will be available for review by the sponsor upon request.

For each batch of unit doses, the empty used unit dose containers will be discarded upon satisfactory completion of the compliance and accountability procedures. Any unused assembled unit doses will be retained until completion of the study.

At the completion of the study, all unused supplies will be returned to the sponsor or disposed of by the study site, per the sponsor's written instructions.

6. CONCOMITANT THERAPIES AND OTHER RESTRICTIONS

6.1. Permitted Concomitant Therapies

Any medication (including over-the-counter or prescription medicines, vitamins, and/or herbal supplements) that the patient is receiving at the time of enrolment or receives during the study must be recorded along with:

- Reason for use
- Dates of administration including start and end dates
- Dosage information including dose and frequency.

The medical monitor should be contacted if there are any questions regarding concomitant or prior therapy.

The following therapies are permitted during the study:

- Supportive therapy considered necessary for the patient's welfare at the discretion of the investigator
- Palliative radiotherapy for the control of bone pain or other symptoms; irradiated lesions will not be evaluable for response
- Hormone-replacement therapy
- Oral, implantable, transdermal, injectable, or intrauterine hormonal contraceptives
- Androgen deprivation therapy for prostate cancer patients.

6.2. Restricted Concomitant Therapies

Patients should not receive any vaccine within 28 days before the first dose of study drug through Week 8.

Patients should avoid receiving the following therapies from screening through the EOS visit:

- Chemotherapy or any other anticancer or biological therapy
- Cancer immunotherapy
- Hormonal therapy that is not considered part of the patient's routine therapy
- Any other investigational product
- Radiotherapy with the exception of palliative radiotherapy
- Systemic glucocorticoids unless required to treat an AE; patients are permitted to use topical, ocular, intra-articular, intranasal, and inhalational corticosteroids (with minimal systemic absorption); adrenal replacement steroid doses > 10 mg daily prednisone may be permitted after consultation with the medical monitor

- Herbal remedies with immunostimulating properties or known to potentially interfere with major organ function.

7. STUDY ASSESSMENTS AND PROCEDURES

Study procedures and their timing are summarised in the schedule of assessments in Appendix 6. As protocol waivers or exemptions are not allowed, with the exception of immediate safety concerns, these should be discussed with the medical monitor immediately upon occurrence or awareness to determine if the patient should continue or discontinue the study drug. Adherence to the study design requirements, including those specified in the schedule of assessments, is essential and required for study conduct.

All screening evaluations must be completed and reviewed to confirm that potential patients meet all eligibility criteria. The investigator will maintain a screening log to record details of all patients screened and to confirm eligibility or record reasons for screening failure, as applicable. Procedures conducted as part of the patient's routine clinical management (e.g., blood count) and obtained before signing the ICF may be utilised for screening or baseline purposes provided the procedure met the protocol-specified criteria and was performed within the timeframe defined in the schedule of assessments.

The maximum amount of blood collected from each patient over the duration of the study, including any extra assessments that may be required, will not exceed 500 mL. Repeat or unscheduled samples may be taken for safety reasons or for technical issues with the samples.

7.1. Efficacy Assessments

7.1.1. Immune Response

Blood samples will be collected at the times indicated in the schedule of assessments in Appendix 6 and sent to the central laboratory for evaluation of T-cell responses utilising ELISpot and antibody responses using ELISA. Procedures for collection, processing, and shipping of blood samples will be detailed in a separate document.

7.1.2. Tumour Response

Tumour response will be assessed using RECIST v1.1. Contrast-enhanced CT (unless contra-indicated) or MRI will be performed at the times indicated in the schedule of assessments in Appendix 6 and as clinically indicated. The CT/MRI will include the following:

- Chest and abdomen for NSCLC patients
- Chest (in those patients with disease in the chest or upper abdomen lymphadenopathy at baseline or in case of respiratory symptoms and if considered necessary by the investigator), abdomen, and pelvis for ovarian cancer patients
- Chest, abdomen, and pelvis for prostate cancer patients.

Imaging will also include radionuclide bone scan for prostate cancer patients. Any additional scans or radiological assessments that are needed to document tumour sites relevant to the particular tumour at baseline must be performed. Patients with known or suspected brain

metastases should have CT or MRI scans of the brain. Any NSCLC or ovarian cancer patients with bone lesions may have a bone scan during screening and repeat bone scans performed as clinically indicated.

Each lesion that is measured at baseline must be measured by the same method throughout the study so that the comparison is consistent. The CT/MRI scan will be read at the site and tumour response will be assessed using RECIST v1.1 as outlined in Appendix 1. The data will be recorded in the patient's source documentation and eCRF.

For prostate cancer patients, the methodologies described by the PCWG3 will be used to assess bone lesions (15). Changes in bone scans will be recorded as improved or stable (no new lesions) or worse (new lesions).

7.1.3. Tumour Markers

7.1.3.1. Cancer Antigen-125

Blood samples will be collected from ovarian cancer patients at the times indicated in the schedule of assessments in Appendix 6 for CA-125 measurement by the local laboratory.

7.1.3.2. Prostate-specific Antigen and Total Alkaline Phosphatase

Blood samples will be collected from prostate cancer patients at the times indicated in the schedule of assessments in Appendix 6 for PSA and ALP measurement by the local laboratory.

7.1.4. Tumour Survivin Expression

A suitable archival sample should be provided during screening. If multiple biopsies are available, samples from the most recent biopsy/surgery should be collected. The sample should have been fixed in formalin and embedded in paraffin. If the archival paraffin block cannot be sent for analysis, the site should make 5 unstained slides (preferably 5 µm sections). Procedures for processing and shipping tissue samples will be detailed in a separate document.

A validated immunohistochemistry assay will be used by the central laboratory to determine survivin expression levels in the tumour.

7.2. Safety and Tolerability Assessments

Reference safety information (RSI) details can be found in the separate IB.

7.2.1. Adverse Events

Adverse event definitions, assignment of severity and causality, and procedures for reporting SAEs are detailed in Appendix 2. Any clinically significant abnormality in clinical laboratories, vital signs measurements, 12-lead electrocardiograms (ECG)s, physical examinations, or Eastern Cooperative Oncology Group (ECOG) performance status (Section 7.2.6) should be recorded as an AE. Adverse events include AEs occurring from overdose of a sponsor study drug whether accidental or intentional (i.e., a dose higher than that prescribed

by a health care professional for clinical reasons) or incorrect administration of a sponsor study drug.

The condition of each patient will be monitored from the time of signing the ICF to final discharge from the study. Patients will be observed for any signs or symptoms and asked about their condition by open questioning, such as “How have you been feeling since you were last asked?”, at least once each day while resident at the study site and at each study visit. Patients will also be encouraged to spontaneously report AEs occurring at any other time during the study.

Any AEs and remedial action required will be recorded in the patient’s source data. The nature, time of onset, duration, and severity will be documented, together with an investigator’s (or designee’s) opinion of the relationship to study drug.

Adverse events recorded during the course of the study will be followed up, where possible, until resolution or until the unresolved AEs are judged by the investigator (or designee) to have stabilised.

Progression of disease is an endpoint of this study and should not be recorded as an AE, as well as events that are unequivocally related to disease progression (except events of death due to disease progression). Progressive disease will be captured on the tumour response eCRFs.

7.2.1.1. Adverse Events of Special Interest

Adverse events of special interest (AESIs) for study drug include the following:

- Significant local or systemic injection site reactions such as anaphylaxis, angioedema, bronchospasm, systemic rash, systemic or local hives.
- Hypersensitivity reactions that are considered by the investigator to be allergic in nature and severe nonallergic injection reactions.

These events of special interest will need to be expeditiously reported to the sponsor irrespective of regulatory seriousness criteria. These should be reported as a syndrome (e.g., hypersensitivity reaction) rather than as individual signs and symptoms (e.g., chills, fever).

Mild or moderate, nonallergic, local injection reactions do not need to be reported in an expedited manner if only minimal medical treatment is required (e.g., ice, paracetamol, or nonsteroidal anti-inflammatory drugs) and if they do not interfere considerably with the patient’s activities. These may include local discomfort; uncomplicated ecchymosis; bleeding; or small areas of delayed redness, induration, or swelling at the injection site. These will be reported as AEs in the eCRF. However, injection site reactions judged to be severe should be treated as AESIs and should be reported to the sponsor within 24 hours even if no SAE conditions are met.

7.2.1.2. Abnormal Clinical Laboratory Values

Not every laboratory abnormality qualifies as an AE. A laboratory test result should be reported as an AE if it meets any of the following criteria:

- Is a DLT
- Is accompanied by clinical symptoms
- Results in a change in study treatment (e.g., treatment interruption or treatment discontinuation)
- Results in a medical intervention (e.g., potassium supplementation for hypokalaemia) or a change in concomitant therapy
- Is clinically significant in the investigator's judgement.

Medical and scientific judgement should be exercised in deciding whether an isolated laboratory abnormality should be classified as an AE.

If a clinically significant laboratory abnormality is a sign of a disease or syndrome, only the diagnosis should be recorded on the AE eCRF. If a clinically significant laboratory abnormality is not a sign of a disease or syndrome, the abnormality itself should be recorded on the AE eCRF.

Observations of the same clinically significant laboratory abnormality from visit to visit should only be recorded once on the AE eCRF.

7.2.2. Clinical Laboratory Evaluations

Blood and urine samples will be collected for clinical laboratory evaluations at the times indicated in the schedule of assessments in Appendix 6. Clinical laboratory evaluations are listed in Appendix 3. Additional clinical laboratory evaluations may be performed at other times if judged to be clinically appropriate or if the ongoing review of the data suggests a more detailed assessment of clinical laboratory safety evaluations is required.

For women of childbearing potential, a urine pregnancy test will be performed at the times indicated in the schedule of assessments in Appendix 6.

An investigator (or designee) will perform a clinical assessment of all clinical laboratory data.

7.2.3. Vital Signs

Seated blood pressure, seated pulse rate, respiratory rate, and oral body temperature will be assessed at the times indicated in the schedule of assessments in Appendix 6. Vital signs may also be performed at other times if judged to be clinically appropriate or if the ongoing review of the data suggests a more detailed assessment of vital signs is required.

Day 1 blood pressure, pulse rate, and respiratory rate will be measured predose in triplicate at approximately 2-minute intervals. The median value will be used as the baseline value in the data analysis. All subsequent measurements will be performed one time and repeated once if outside the relevant clinical reference ranges. Oral body temperature will be measured singly.

Patients must be seated for at least 5 minutes before blood pressure and pulse rate measurements.

7.2.4. Electrocardiogram

Resting 12-lead ECGs will be recorded after the patient has been supine and at rest for at least 5 minutes at the times indicated in the schedule of assessments in Appendix 6. A 12-lead ECG will be repeated once if either of the following criteria apply:

- QT interval corrected for heart rate using Fridericia's method (QTcF) value > 500 ms
- QTcF change from the baseline (screening) is > 60 ms.

Additional 12-lead ECGs may be performed at other times if judged to be clinically appropriate or if the ongoing review of the data suggests a more detailed assessment of ECGs is required. The investigator (or designee) will perform a clinical assessment of each 12-lead ECG.

Screening 12-lead ECGs will be measured in triplicate at approximately 2-minute intervals. The mean value will be used as the baseline value in the data analysis. All subsequent measurements will be performed one time and repeated once if outside the relevant clinical reference ranges.

7.2.5. Physical Examination

A full physical examination will be performed at the timepoints specified in the schedule of assessments in Appendix 6.

7.2.6. Eastern Cooperative Oncology Group (ECOG) Performance Score

ECOG performance status as specified in Table 3 will be evaluated at the times indicated in the schedule of assessments in Appendix 6.

Table 3: Eastern Cooperative Oncology Group Performance Status Scale

Grade	Description
0	Fully active, able to carry on all predisease performance without restriction.
1	Restricted in physically strenuous activity, but ambulatory and able to carry out work of a light or sedentary nature (e.g., light house work, office work).
2	Ambulatory and capable of all self-care, but unable to carry out any work activities. Up and about more than 50% of waking hours.
3	Capable of only limited self-care, confined to bed or chair more than 50% of waking hours.
4	Completely disabled. Cannot carry on any self-care. Totally confined to bed or chair.
5	Dead.

8. SAMPLE SIZE AND DATA ANALYSIS

8.1. Determination of Sample Size

Phase 1a: No formal sample size calculation has been performed. The sample size is based primarily upon clinical considerations and is typical for FIH, oncologic, dose-escalation studies.

Phase 1b: The sample size is based primarily upon clinical considerations. However, in terms of the overall assessment of immune response and RECIST response, 24 patients overall will allow for evaluation of response rates with an approximate precision of 10% in the worst-case scenario.

8.2. Analysis Populations

8.2.1. Safety Evaluable Population

The safety evaluable population is defined as all patients in either Phase who received OVM-200 regardless of the duration of treatment received. Safety parameters and efficacy parameters such as PFS; OS; and CA-125, PSA, and ALP responses will be assessed in this population.

8.2.2. Immune Response Evaluable Population

The immune response evaluable population is defined as all patients who received OVM-200 in either Phase and had at least 1 predose and 1 postbaseline sample taken to assess immune response. Immune response as measured by ELISpot and ELISA will be assessed in this population.

8.2.3. Response Evaluation Criteria in Solid Tumour Response Evaluable Population

The RECIST response evaluable population is defined as all patients who received OVM-200 in either Phase and have at least 1 postbaseline assessment for the RECIST response. The ORR, TTR, and DoR will be assessed in this population.

8.3. General Considerations

Demographic data and disease-related characteristics for Phases 1a and 1b will be summarised overall and by therapeutic indication (NSCLC, ovarian cancer, and prostate cancer) using descriptive statistics as appropriate. Categorical variables will be summarised by frequency counts and percentages for each category. Continuous variables will be summarised by the number of observations, mean, median, standard deviation, minimum, and maximum. All patient data will be summarised.

8.4. Efficacy Analyses

8.4.1. Immune Response

An immune response will be considered if using geometric means across 3 patients at each level a difference between preimmunisation and postimmunisation is calculated to be

2 standard deviations or more. In Phase 1a, immune response will be summarised by dose and timepoint using frequency counts and percentages. Immune response will be presented by therapeutic indication (NSCLC, ovarian cancer, and prostate cancer) as well as overall using methodology akin to that used in basket study designs (1). Immune response may also be analysed supportively in a Bayesian manner; this would entail the Bayesian combination, by cohort, of responses observed in Phase 1a with corresponding responses observed in Phase 1b.

8.4.2. Objective Response Rate

The ORR is defined as patients with an investigator-assessed response of complete response (CR) or partial response (PR) as per RECIST. In Phase 1a, ORR will be summarised by visit and dose using frequency counts and percentages. The ORR may be analysed supportively in a Bayesian manner; this would entail the Bayesian combination, by cohort, of responses observed in Phase 1a with corresponding responses observed in Phase 1b.

8.4.3. Time to Response

The TTR is defined for patients with CR or PR as the time from the first dose of OVM-200 to the first objective response (CR or PR). Patients who do not experience CR or PR will be censored at the time of their last RECIST assessment. The TTR may be displayed graphically using Kaplan-Meier plots by cohort utilising the combined data from Phases 1a and 1b. Median [95% Confidence Interval (CI)] TTR will be reported, based on the Kaplan-Meier estimate, where appropriate.

8.4.4. Duration of Response

The DoR is defined for patients with an investigator-assessed response of CR or PR as the time from the date of objective response to the first of disease progression (bone or soft tissue) or death. Patients who do not experience disease progression or death will be censored at the time of their last RECIST assessment. If 5 or more patients experience disease progression or death in Phases 1a and 1b combined, the DoR will be displayed graphically using Kaplan-Meier plots by cohort utilising the combined data from Phases 1a and 1b. Median (95% CI) DoR will be reported based on the Kaplan-Meier estimate, where appropriate.

8.4.5. Overall Survival

The OS is defined as the time from study entry to death due to any cause. Patients who are alive will be censored at the time of their last site contact. For patients who complete the study, this will be administrative censoring at Week 24. The OS may be displayed graphically using Kaplan-Meier plots by cohort utilising the combined data from Phases 1a and 1b. Median (95% CI) OS will be reported, based on the Kaplan-Meier estimate, where appropriate.

8.4.6. Progression-free Survival

The PFS is defined as the time from study entry to disease progression (bone or soft tissue) or death. Patients who do not experience disease progression or death will be censored at the time of their last RECIST assessment. The PFS may be displayed graphically using

Kaplan-Meier plots by cohort utilising the combined data from Phases 1a and 1b. Median (95% CI) PFS will be reported, based on the Kaplan-Meier estimate, where appropriate.

8.4.7. Cancer Antigen-125 Response

In ovarian cancer patients, CA-125 response will be based on the GCIC (16). Patients will be evaluable for CA-125 response if all the following are met:

- A pretreatment CA-125 level (taken within 2 weeks prior to starting study drug) is $\geq 2 \times$ ULN
- There is no more than a 10% reduction in CA-125 between the 2 pretreatment measurements
- The same assay method is used for each sample from the same patient.

A response according to CA-125 will be considered to have occurred if there is ≥ 50% reduction in CA-125 levels from the last pretreatment sample. The CA-125 response will be summarised by visit using frequency counts and percentages.

The CA-125 measures over time will be displayed graphically for each patient using spaghetti plots and maximal change (rise or fall) based on the overall visit responses will be tabulated and displayed using a waterfall plot.

8.4.8. Prostate-specific Antigen and Total Alkaline Phosphatase Response

In prostate cancer patients, the change from baseline (rise or fall, on the ratio scale) in PSA and ALP will be tabulated by visit, and the maximal change (rise or fall) based on the overall visit assessments will be tabulated and displayed using a waterfall plot.

A PSA response will be defined as a decrease of ≥ 50% from baseline values, and an ALP response will be defined as a decrease of > 30% from baseline values. The PSA and ALP response will be tabulated by visit using frequency counts and percentages.

Where data are available, an estimated PSADT before study entry will be determined and tabulated with estimated on-study PSADT. Longitudinal PSA measures over time will be displayed graphically for each patient using spaghetti plots.

8.4.9. Relationship to Survivin Expression

The relationship between survivin expression and immune response, objective tumour response (based on RECIST), and tumour marker response (CA-125 [ovarian cancer patients; Section 8.4.7], and PSA and ALP [prostate cancer patients; Section 8.4.8]) will be explored via logistic regression analyses. The log(postdose/baseline) survivin expression will be included as the independent variable in logistic regression analyses. For PSA and ALP, the log odds of response will be characterised as a function of log(post/baseline) expression level.

Cox proportional hazards model may be used to explore the relationship between TTR, DoR, OS, and PFS and baseline survivin expression.

8.5. Safety Analysis

Safety data will be summarised by phase, cohort, and overall using descriptive statistics. No formal statistical analysis of safety data is planned. Adverse event terms will be coded using MedDRA Version 24.0 and summarised by body system, preferred term, severity, and relationship to study drug.

The number and percentage of patients with AEs will be summarised for the following categories:

- Any AEs
- Any AEs causally related to study drug
- Any AEs with outcome of death
- Any AEs leading to discontinuation of study drug
- Any SAEs.

Clinical laboratory assessments will be converted to standard units and summarised by maximum National Cancer Institute Common Terminology Criteria for Adverse Events v5.0 severity grade. Vital signs and 12-lead ECGs will be summarised using descriptive statistics. Physical examination results and ECOG performance status (Section 7.2.6) will be listed.

8.6. Interim Analysis

No formal interim analyses are planned for this study.

9. REFERENCES

1. Berry SM, Broglio KR, Groshen S, Berry DA. Bayesian hierarchical modeling of patient subpopulations: efficient designs of Phase II oncology clinical trials. Clin Trials. 2013;10(5):720-34.

2. Garg H, Suri P, Gupta JC, Talwar GP, Dubey S. Survivin: a unique target for tumor therapy. Cancer Cell Int. 2016;16:49.

3. Wheatley SP, Altieri DC. Survivin at a glance. Journal of Cell Science. 2019;132(7).

4. Cai L, Zhang J, Zhu R, Shi W, Xia X, Edwards M, et al. Protective cellular immunity generated by cross-presenting recombinant overlapping peptide proteins. Oncotarget. 2017;8(44):76516-24.

5. Kartikasari AER, Prakash MD, Cox M, Wilson K, Boer JC, Cauchi JA, et al. Therapeutic Cancer Vaccines—T Cell Responses and Epigenetic Modulation. Front Immunol. 2019;9(3109).

6. Roche PA, Furuta K. The ins and outs of MHC class II-mediated antigen processing and presentation. Nat Rev Immunol. 2015;15(4):203-16.

7. Zhang H, Hong H, Li D, Ma S, Di Y, Stoten A, et al. Comparing pooled peptides with intact protein for accessing cross-presentation pathways for protective CD8+ and CD4+ T cells. J Biol Chem. 2009;284(14):9184-91.

8. van Doorn E, Liu H, Huckriede A, Hak E. Safety and tolerability evaluation of the use of Montanide ISA™51 as vaccine adjuvant: A systematic review. Hum Vaccin Immunother. 2016;12(1):159-69.

9. McNeil MM. Vaccine-Associated Anaphylaxis. Curr Treat Options Allergy. 2019;6(3):297-308.

10. Su JR, Moro PL, Ng CS, Lewis PW, Said MA, Cano MV. Anaphylaxis after vaccination reported to the Vaccine Adverse Event Reporting System, 1990-2016. J Allergy Clin Immunol. 2019;143(4):1465-73.

11. Phillips A, Hickie M, Totterdell J, Brotherton J, Dey A, Hill R, et al. Adverse events following HPV vaccination: 11 years of surveillance in Australia. Vaccine. 2020;38(38):6038-46.

12. Rampling R, Peoples S, Mulholland PJ, James A, Al-Salihi O, Twelves CJ, et al. A Cancer Research UK First Time in Human Phase I Trial of IMA950 (Novel Multipeptide Therapeutic Vaccine) in Patients with Newly Diagnosed Glioblastoma. Clin Cancer Res. 2016;22(19):4776-85.

13. Amato RJ, Shetty A, Lu Y, Ellis R, Low PS. A phase I study of folate immune therapy (EC90 vaccine administered with GPI-0100 adjuvant followed by EC17) in patients with renal cell carcinoma. J Immunother. 2013;36(4):268-75.

14. Golden DB. Patterns of anaphylaxis: acute and late phase features of allergic reactions. Novartis Found Symp. 2004;257:101-10; discussion 10-5, 57-60, 276-85.

15. Scher HI, Morris MJ, Stadler WM, Higano C, Basch E, Fizazi K, et al. Trial Design and Objectives for Castration-Resistant Prostate Cancer: Updated Recommendations From the Prostate Cancer Clinical Trials Working Group 3. J Clin Oncol. 2016;34(12):1402-18.

16. Rustin GJ, Vergote I, Eisenhauer E, Pujade-Lauraine E, Quinn M, Thigpen T, et al. Definitions for response and progression in ovarian cancer clinical trials incorporating RECIST 1.1 and CA 125 agreed by the Gynecological Cancer Intergroup (GCIG). Int J Gynecol Cancer. 2011;21(2):419-23.

17. Brunsvig PF, Guren TK, Nyakas M, Steinfeldt-Reisse CH, Rasch W, Kyte JA, et al. Long-Term Outcomes of a Phase I Study With UV1, a Second Generation Telomerase Based Vaccine, in Patients With Advanced Non-Small Cell Lung Cancer. Front Immunol. 2020;11:572172-.

18. Jaiswal PK, Goel A, Mittal RD. Survivin: A molecular biomarker in cancer. Indian J Med Res. 2015;141(4):389-97.

19. Fenstermaker RA, Ciesielski MJ, Qiu J, Yang N, Frank CL, Lee KP, et al. Clinical study of a survivin long peptide vaccine (SurVaxM) in patients with recurrent malignant glioma. Cancer Immunol Immunother. 2016;65(11):1339-52.

20. NCT01543464. Peptide Vaccine and Temozolomide for Metastatic Melanoma Patients [Available from: https://ClinicalTrials.gov/show/NCT01543464.

21. NCT01784913. A Phase I/IIa Study of UV1 Vaccine in Patients With Prostate Cancer [Available from: https://ClinicalTrials.gov/show/NCT01784913.

22. NCT00005629. Vaccine Therapy in Treating Patients With Liver Cancer [Available from: https://ClinicalTrials.gov/show/NCT00005629.

23. NCT00257738. 0804 GCC: MAGE-A3/HPV 16 Vaccine for Squamous Cell Carcinoma of the Head and Neck [Available from: https://ClinicalTrials.gov/show/NCT00257738.

24. Lilleby W, Gaudernack G, Brunsvig PF, Vlatkovic L, Schulz M, Mills K, et al. Phase I/IIa clinical trial of a novel hTERT peptide vaccine in men with metastatic hormone-naive prostate cancer. Cancer Immunol Immunother. 2017;66(7):891-901.

25. Radunskaya A, Hook S. Modeling the Kinetics of the Immune Response. In: d'Onofrio A, Cerrai P, Gandolfi A, editors. New Challenges for Cancer Systems Biomedicine. Milano: Springer Milan; 2012. p. 267-82.

26. NCT00488592. Peptide Vaccinations to Treat Patients With Low-Risk Myeloid Cancers [Available from: https://ClinicalTrials.gov/show/NCT00488592.

27. NCT02455557. SurVaxM Vaccine Therapy and Temozolomide in Treating Patients With Newly Diagnosed Glioblastoma [Available from: https://ClinicalTrials.gov/show/NCT02455557.

28. Muderspach L, Wilczynski S, Roman L, Bade L, Felix J, Small LA, et al. A phase I trial of a human papillomavirus (HPV) peptide vaccine for women with high-grade cervical and vulvar intraepithelial neoplasia who are HPV 16 positive. Clin Cancer Res. 2000;6(9):3406-16.

29. Murahashi M, Hijikata Y, Yamada K, Tanaka Y, Kishimoto J, Inoue H, et al. Phase I clinical trial of a five-peptide cancer vaccine combined with cyclophosphamide in advanced solid tumors. Clin Immunol. 2016;166-167:48-58.

30. European Commission E. EudraLex - Volume 4 - Good Manufacturing Practice (GMP) guidelines [Available from: https://ec.europa.eu/health/documents/eudralex/vol-4/.

31. Eisenhauer EA, Therasse P, Bogaerts J, Schwartz LH, Sargent D, Ford R, et al. New response evaluation criteria in solid tumours: revised RECIST guideline (version 1.1). Eur J Cancer. 2009;45(2):228-47.

10. APPENDICES

Appendix 1: Response Evaluation Criteria in Solid Tumours v1.1

Based on the following publication: (31)

Measurable Disease

Tumour lesions: Must be accurately measured in at least 1 dimension (longest diameter in the plane of measurement is to be recorded) with a minimum size of:

- 10 mm by computed tomography (CT) scan (CT scan slice thickness recommended to be ≤ 5 mm) or magnetic resonance imaging (MRI) scan (no less than double the slice thickness and ≥ 10 mm)
- 10 mm calliper measurement by clinical examination (when superficial)
- 20 mm by chest X-ray (if clearly defined and surrounded by aerated lung).

Malignant lymph nodes: To be considered pathologically enlarged and measurable, a lymph node must be ≥ 15 mm in the short axis when assessed by CT/MRI scan (scan slice thickness recommended to be no greater than 5 mm). At baseline and postdose, only the short axis will be measured and followed.

Nonmeasurable Disease

All other lesions, including small lesions (longest diameter < 10 mm or pathological lymph nodes ≥ 10 to < 15 mm in the short axis) as well as truly nonmeasurable lesions are considered nonmeasurable lesions. Leptomeningeal disease, ascites, pleural or pericardial effusion, lymphangitic involvement of skin or lung, inflammatory breast disease, and abdominal mass/abdominal organomegaly identified by physical examination that is not measurable by reproducible imaging techniques are all nonmeasurable.

Target Lesions

All measurable lesions up to a maximum of 2 lesions per organ and 5 lesions in total should be identified as target lesions and be recorded and measured at baseline. Target lesions should be selected on the basis of their size (lesions with the longest diameter), be representative of all involved organs, and in addition should be those that lend themselves to reproducible repeated measurements. The sum of the diameters (longest diameter for nonnodal lesions, short axis for nodal lesions) of all target lesions will be calculated and reported as the baseline sum of diameters. If lymph nodes are included, only the short axis is added to the sum.

After baseline, a value should be provided on the electronic case report form (eCRF) for all identified target lesions for each assessment, even if very small. If extremely small and faint lesions cannot be accurately measured, but are deemed to be present, a default value of 5 mm may be used. If lesions are too small to measure and indeed are believed to be absent, a default value of 0 mm may be used.

Nontarget Lesions

All other lesions (or sites of disease), including pathological lymph nodes, should be identified as nontarget lesions and should also be recorded at baseline. Measurements are not required, and these lesions should be noted and followed as "present," "absent," or in rare cases "unequivocal progression." It is possible to record multiple nontarget lesions involving the same organ as a single item on the eCRF (e.g., "multiple enlarged pelvic lymph nodes" or "multiple liver metastases").

Evaluation of Target Lesions

Target lesions will be assessed as follows:

- Complete response (CR): disappearance of all target lesions. Any pathological lymph nodes (whether target or nontarget) must have reduction in short axis to < 10 mm
- Partial response (PR): at least a 30% decrease in the sum of diameters of target lesions, taking as reference the baseline sum of diameters
- Progressive disease (PD): at least a 20% increase in the sum of diameters of target lesions, taking as reference the smallest sum on study (this includes the baseline sum if that is the smallest on study). In addition to the relative increase of 20%, the sum must also demonstrate an absolute increase of at least 5 mm. (Note: The appearance of one or more new lesions is also considered progression.)
- Stable disease (SD): neither sufficient shrinkage to qualify for PR nor sufficient increase to qualify for PD, taking as reference the smallest sum of diameters while on study.

Evaluation of Nontarget Lesions

Nontarget lesions will be assessed as follows:

- CR: disappearance of all nontarget lesions and normalisation of tumour marker level. All lymph nodes must be nonpathological in size (< 10 mm short axis)
- Non-CR/Non-PD: persistence of one or more nontarget lesion(s) and/or maintenance of tumour marker level above the normal limits
- PD: unequivocal progression of existing nontarget lesions (Note: The appearance of one or more new lesions is also considered progression).

When the patient also has measurable disease, to achieve "unequivocal progression" on the basis of the nontarget disease, there must be an overall level of substantial worsening in nontarget disease such that, even in the presence of SD or PR in target disease, the overall tumour burden has increased sufficiently to merit discontinuation of therapy. A modest "increase" in the size of one or more nontarget lesions is usually not sufficient to quality for unequivocal progression status. Therefore, the designation of overall progression solely on the basis of change in nontarget disease in the face of SD or PR of target disease will be extremely rare.

Overall Response

Overall response should be assessed according to the table below for patients with target lesions.

Evaluation of Overall Response for Patients with Measurable Disease at Baseline

Target Lesions Response	Nontarget Lesions Response	New Lesions	Overall Response
CR	CR	No	CR
CR	Non-CR/Non-PD	No	PR
CR	NE	No	PR
PR	Non-PD or NE	No	PR
SD	Non-PD or NE	No	SD
Not all evaluated	Non-PD	No	NE
PD	Any	Yes or No	PD
Any	PD	Yes or No	PD
Any	Any	Yes	PD

Abbreviations: CR = complete response; PD = progressive disease; PR = partial response; SD = stable disease; NE = not evaluable.

Appendix 2: Adverse Event Reporting

Definitions

An adverse event (AE) is any untoward medical occurrence in a patient or clinical investigation subject administered a pharmaceutical product, which does not necessarily have a causal relationship with this treatment. An AE can therefore be any unfavourable and/or unintended sign (including a clinically significant abnormal laboratory finding), symptom, or disease temporally associated with the use of a study treatment, whether or not related to the study treatment.

Assessment of Severity

Whenever possible, intensity will be classified according to the criteria provided by the National Cancer Institute Common Terminology Criteria for Adverse Events (NCI CTCAE) v5.0. For AEs that are not specifically listed in the NCI CTCAE, the AE should be graded as follows:

- **Grade 1:** mild; asymptomatic or mild symptoms; clinical or diagnostic observations only; intervention not indicated
- **Grade 2:** moderate; minimal, local, or noninvasive intervention indicated; limiting age-appropriate instrumental activities of daily living (ADL)
- **Grade 3:** severe or medically significant, but not immediately life-threatening; hospitalisation or prolongation of hospitalisation indicated; disabling; limiting self-care ADL
- **Grade 4:** life-threatening consequences; urgent intervention indicated
- **Grade 5:** death related to AE.

Relationship to Study Drug

The investigator will make a determination of the relationship of the AE to the study drug using a 4-category system according to the following guidelines:

- **Not related:** The AE is definitely caused by the patient's clinical state or the study procedure/conditions.
- **Unlikely related:** The temporal association between the AE and the drug is such that the drug is not likely to have any reasonable association with the AE.
- **Possibly related:** The AE follows a reasonable temporal sequence from the time of drug administration but could have been produced by the patient's clinical state or the study procedures/conditions.
- **Related:** The AE follows a reasonable temporal sequence from administration of the drug, abates upon discontinuation of the drug, follows a known or hypothesised cause-effect relationship, and (if appropriate) reappears when the drug is reintroduced.

Action Taken for Adverse Events

The investigator or designee will record the action taken for the AE in the electronic case report form (eCRF). Actions taken will include:

- **Dose increased:** The medication schedule was modified by addition, either by changing the frequency, strength, or amount.
- **Dose not changed:** The medication schedule was not changed.
- **Dose reduced:** The medication schedule was modified by subtraction, either by changing the frequency, strength, or amount.
- **Drug interrupted:** The medication schedule was modified by temporarily terminating the prescribed regimen of medication.
- **Drug withdrawn:** The medication schedule was modified through termination of the prescribed regimen of medication.
- **Not applicable**
- **Unknown**

Follow up of Adverse Events

Every reasonable effort will be made to follow up with patients who have AEs. Any patient who has an ongoing AE that is possibly related or related to the investigational medicinal product (IMP) or study procedures at the end-of-study (EOS) visit will be followed up, where possible, until resolution or until the unresolved AE is judged by the investigator (or designee) to have stabilised. Any patient who has an ongoing AE that is not related or unlikely related to the IMP or study procedures at the EOS visit can be closed out as ongoing at the investigator's discretion.

Adverse Drug Reactions

All noxious and unintended responses to an IMP (ie, where a causal relationship between an IMP and an AE is at least a reasonable possibility) related to any dose should be considered adverse drug reactions.

For marketed medicinal products, a response to a drug that is noxious and unintended and that occurs at doses normally used in man for prophylaxis, diagnosis, or therapy of diseases or for modification of physiological function is to be considered an adverse drug reaction.

An unexpected adverse drug reaction is defined as an adverse reaction, the nature or severity of which is not consistent with the applicable product information (e.g., investigator's brochure for an unapproved IMP).

Serious Adverse Events

A serious AE (SAE) is defined as any untoward medical occurrence that at any dose either:

- Results in death
- Is life-threatening
- Requires inpatient hospitalisation or prolongation of existing hospitalisation
- Results in persistent or significant disability/incapacity (disability is defined as a substantial disruption of a person's ability to conduct normal life functions)
- Results in a congenital anomaly/birth defect
- Results in an important medical event (see below).

Important medical events that may not result in death, be life-threatening, or require hospitalisation may be considered SAEs when, based upon appropriate medical judgement, they may jeopardise the patient and may require medical or surgical intervention to prevent one of the outcomes listed in this definition.

Instances of death or congenital abnormality, if brought to the attention of the investigator at any time after cessation of the study treatment and considered by the investigator to be possibly related to the study treatment, will be reported to the sponsor.

Definition of Life-threatening

An AE is life-threatening if the patient was at immediate risk of death from the event as it occurred (ie, does not include a reaction that might have caused death if it had occurred in a more serious form). For instance, drug-induced hepatitis that resolved without evidence of hepatic failure would not be considered life-threatening even though drug-induced hepatitis can be fatal.

Definition of Hospitalisation

Adverse events requiring hospitalisation should be considered serious. In general, hospitalisation signifies that the patient has been detained (usually involving an overnight stay) at the hospital or emergency ward for observation and/or treatment that would not have been appropriate at the site. When in doubt as to whether hospitalisation occurred or was necessary, the AE should be considered as serious.

Hospitalisation for elective surgery or routine clinical procedures, which are not the result of an AE, need not be considered AEs and should be recorded on a clinical assessment form and added to the eCRF. If anything untoward is reported during the procedure, this must be reported as an AE and either 'serious' or 'nonserious' attributed according to the usual criteria.

Serious Adverse Event Reporting

Covance Patient Safety Solutions (PSS) Europe, Maidenhead, United Kingdom, are responsible for coordinating the reporting of SAEs in accordance with the European Directive 2001/20/EC.

The investigator will complete an SAE report form and forward it by facsimile or email to PSS and the sponsor immediately (within 24 hours) upon becoming aware of an SAE.

The responsibilities of Covance PSS include the following:

- Prepare an AE reporting plan prior to the start of the study. Where this plan differs from the applicable site standard operating procedure on SAE reporting, the safety management plan will always take precedence
- Receive and review SAE report forms from the site and inform the sponsor of the SAE within 1 working day of the initial notification to PSS. Patient Safety Solutions will delete any information from the SAE report forms that may identify the patient
- Write case narratives and enter the case into Covance's safety database as defined in the AE reporting plan
- Produce appropriate reports of all Suspected Unexpected Serious Adverse Reactions and forward to the ethics committee (EC), Medicines and Healthcare Products Regulatory Agency, principal investigator, and sponsor within the timeframes stipulated in the Clinical Trials Directive Guideline (ENTR/CT 3).

The responsibility for reporting SAEs will be transferred to the sponsor 28 days after the end of the study.

Pregnancy

Pregnancy (maternal or paternal exposure to study treatment) does not meet the definition of an AE. However, to fulfil regulatory requirements any pregnancy should be reported following the SAE process to collect data on the outcome for both mother and foetus.

Overdose

Study drug overdose is the accidental or intentional use of the drug in an amount higher than the dose being studied. An overdose or incorrect administration of study drug is not an AE unless it results in untoward medical effects.

Any study drug overdose or incorrect administration of study drug should be noted on the Study Drug Administration eCRF page.

All AEs associated with an overdose or incorrect administration of study drug should be recorded on the AE eCRF page. If the associated AE fulfils serious criteria, the event should be reported to the sponsor immediately (ie, no more than 24 hours after learning of the event).

Appendix 3: Clinical Laboratory Evaluations

Clinical chemistry:	Haematology:	Urinalysis:
Alanine aminotransferase<br>Albumin<br>Aspartate aminotransferase<br>Blood urea nitrogen<br>Calcium<br>Chloride<br>Creatinine<br>Gamma-glutamyl transferase<br>Glucose<br>Inorganic phosphate<br>Potassium<br>Sodium<br>Total alkaline phosphatase<br>Total bilirubin<br>Total protein<br>Uric acid	Haematocrit<br>Haemoglobin<br>Mean cell haemoglobin<br>Mean cell haemoglobin concentration<br>Mean cell volume<br>Platelet count<br>Red blood cell count<br>White blood cell (WBC) count<br>WBC differential:<br>Basophils<br>Eosinophils<br>Lymphocytes<br>Monocytes<br>Neutrophils	Blood<br>Glucose<br>Ketones<br>pH<br>Protein<br>Specific gravity<br>Microscopic examination (if dipstick is abnormal)
**Serology:**	**Other**	
Hepatitis B surface antigen<br>Hepatitis C antibody or ribonucleic acid<br>Human immunodeficiency (HIV-1 and HIV-2) antibodies and p24 antigen (if history)	International normalised ratio<br>Urine pregnancy test (women of childbearing potential)	

Appendix 4: Contraception Guidance

Definitions

Women of Childbearing Potential: premenopausal females who are anatomically and physiologically capable of becoming pregnant following menarche.

Women of Nonchildbearing Potential:

1. **Surgically sterile:** females who are permanently sterile via hysterectomy, bilateral salpingectomy, and/or bilateral oophorectomy by reported medical history and/or medical records. Surgical sterilisation to have occurred a minimum of 6 weeks, or at the investigator's discretion, prior to screening.
2. **Postmenopausal:** females at least 45 years of age with a history of amenorrhoea for 12 months without an alternative medical reason. The amenorrhoea should not be induced by a medical condition, such as anorexia nervosa, hypothyroid disease, or polycystic ovarian disease, or by extreme exercise. It should not be due to concomitant medications that may have induced the amenorrhoea such as oral contraceptives, hormones, gonadotropin-releasing hormones, antioestrogens, or selective oestrogen receptor modulators.

Fertile male: a male that is considered fertile after puberty.

Infertile male: permanently sterile male via bilateral orchiectomy.

Contraception Guidance

Female Patients

Female patients who are of nonchildbearing potential will not be required to use contraception. Female patients of childbearing potential must be willing to use 2 methods (1 primary and 1 secondary method) of birth control from the time of signing the informed consent form (ICF) until 90 days after the end-of-study (EOS) visit. Primary (nonbarrier) methods of contraception include:

- Hormonal injection (as prescribed)
- Combined oral contraceptive pill or progestin/progestogen-only pill (as prescribed)
- Combined hormonal patch (as prescribed)
- Combined hormonal vaginal ring (as prescribed)
- Surgical method performed at least 3 months prior to the screening visit:
  - Bilateral tubal ligation
  - Essure® (hysteroscopic bilateral tubal occlusion) with confirmation of occlusion of the fallopian tubes
- Hormonal implant
- Hormonal or nonhormonal intrauterine device

- Vasectomised male partner (sterilisation performed at least 90 days prior to the screening visit, with verbal confirmation of surgical success, and the sole partner for the female patient).

Secondary (barrier) methods of contraception include:

- Male condom with spermicide
- Female condom with spermicide
- Over-the-counter sponge with spermicide
- Cervical cap with spermicide (as prescribed)
- Diaphragm with spermicide (as prescribed).

Female patients of childbearing potential should refrain from donation of ova from check-in (Day 1) until 90 days after the EOS visit.

Male Patients

Male patients (even with a history of vasectomy) with partners of childbearing potential must use a male barrier method of contraception (i.e., male condom with spermicide) in addition to a second method of acceptable contraception from Day 1 until 90 days after the EOS visit. Acceptable methods of contraception for female partners include:

- Hormonal injection
- Combined oral contraceptive pill or progestin/progestogen-only pill
- Combined hormonal patch
- Combined hormonal vaginal ring
- Surgical method (bilateral tubal ligation or Essure [hysteroscopic bilateral tubal occlusion])
- Hormonal implant
- Hormonal or nonhormonal intrauterine device
- Over-the-counter sponge with spermicide
- Cervical cap with spermicide
- Diaphragm with spermicide.

An acceptable second method of contraception for male patients is vasectomy that has been performed at least 90 days prior to the screening visit, with verbal confirmation of surgical success.

For male patients (even with a history of vasectomy), sexual intercourse with female partners who are pregnant, or breastfeeding should be avoided unless condoms are used from the time of the first dose until 90 days after the EOS visit. Male patients are required to refrain from donation of sperm from Day 1 until 90 days after the EOS visit.

Sexual Abstinence and Same-sex Relationships

Patients who practice true abstinence, because of the patient's lifestyle choice (i.e., the patient should not become abstinent just for the purpose of study participation), are exempt from contraceptive requirements. Periodic abstinence (e.g., calendar, ovulation, symptothermal, postovulation methods) and withdrawal are not acceptable methods of contraception. If a patient who is abstinent at the time of signing the ICF becomes sexually active, they must agree to use contraception as described previously.

For patients who are exclusively in same-sex relationships, contraceptive requirements do not apply. If a patient who is in a same-sex relationship at the time of signing the ICF becomes engaged in a heterosexual relationship, they must agree to use contraception as described previously.

Appendix 5: Regulatory, Ethical, and Study Oversight Considerations

Regulatory and Ethical Considerations

This study will be conducted in accordance with the protocol and with the following:

- Consensus ethical principles derived from international guidelines including the Declaration of Helsinki and Council for International Organizations of Medical Sciences International Ethical Guidelines
- Applicable International Council for/Conference on Harmonisation (ICH) GCP Guidelines
- Applicable laws and regulations.

The protocol, protocol amendments, informed consent form (ICF), investigator's brochure, and other relevant documents must be submitted to an ethics committee (EC) by the investigator and reviewed and approved by the EC before the study is initiated.

Any substantial protocol amendments, likely to affect the safety of the patients or the conduct of the study, will require EC and regulatory authority (as locally required) approval before implementation of changes made to the study design, except for changes necessary to eliminate an immediate hazard to study patients or any nonsubstantial changes, as defined by regulatory requirements.

The investigator will be responsible for the following:

- Providing written summaries of the status of the study to the EC annually or more frequently in accordance with the requirements, policies, and procedures established by the EC
- Notifying the EC of serious adverse events or other significant safety findings as required by EC procedures
- Providing oversight of the conduct of the study at the site and adherence to requirements of 21 Code of Federal Regulations (CFR), ICH guidelines, the EC, European Directive 2001/20/EC for clinical studies (if applicable), and all other applicable local regulations.

Finances and Insurance

Financing and insurance will be addressed in a separate agreement.

Informed Consent

Prior to starting participation in the study, each patient will be provided with a study-specific ICF giving details of the study treatments, procedures, and potential risks of the study. Patients will be instructed that they are free to obtain further information from the investigator (or designee) and that their participation is voluntary and they are free to withdraw from the study at any time. Patients will be given an opportunity to ask questions about the study prior to providing consent for participation.

Patients will be required to sign a statement of informed consent that meets the requirements of local regulations, ICH guidelines, and the EC or study centre, where applicable. The patient will be given a copy of the signed ICF, and the original will be maintained with the patient's records.

Patients must be re-consented to the most current version of the ICF(s) during their participation in the study.

Patient Data Protection

Patients will be assigned a unique identifier and will not be identified by name in electronic case report forms (eCRFs), study-related forms, study reports, or any related publications. Patient and investigator personal data will be treated in compliance with all applicable laws and regulations. In the event the study protocol, study report, or study data are included in a public registry, all identifiable information from individual patients or investigators will be redacted according to applicable laws and regulations.

The patient must be informed that his/her personal study-related data will be used by the sponsor in accordance with local data protection law. The level of disclosure must also be explained to the patient. The patient must also be informed that his/her study-related data may be examined by sponsor or contract research organisation (CRO) auditors or other authorised personnel appointed by the sponsor, by appropriate EC members, and by inspectors from regulatory authorities.

Committees Structure

A safety review committee (SRC) will act in an advisory capacity to recommend the dose escalations for Phase 1a and the dose for Phase 1b. The SRC members will be selected on the basis of relevant experience and understanding of clinical research and the issues specific to the therapeutic area, as well as previous SRC experience. An SRC charter, which includes detailed processes, will be prepared.

Disclosure

All information provided regarding the study, as well as all information collected and/or documented during the course of the study, will be regarded as confidential. The investigator (or designee) agrees not to disclose such information in any way without prior written permission from the sponsor.

Data Quality Assurance

The following data quality steps will be implemented:

- All relevant patient data relating to the study will be recorded on eCRFs unless directly transmitted to the sponsor or designee electronically (e.g., laboratory data). The investigator is responsible for verifying that data entries are accurate and correct by electronically signing the eCRF.
- The investigator must maintain accurate documentation (source data) that supports the information entered in the eCRF.

- The investigator must permit study-related monitoring, audits, EC review, and regulatory agency inspections and provide direct access to source data documents.
- The sponsor or designee is responsible for the data management of this study including quality checking of the data. Predefined agreed risks, monitoring thresholds, quality tolerance thresholds, controls, and mitigation plans will be documented in a project management plan. Additional details of quality checking to be performed on the data may be included in a data management plan.
- Study monitors will perform ongoing source data verification to confirm that data entered into the eCRF by authorised site personnel are accurate, complete, and verifiable from source documents; that the safety and rights of patients are being protected; and that the study is being conducted in accordance with the currently approved protocol and any other study agreements, ICH GCP, and all applicable regulatory requirements.
- Records and documents, including signed ICFs, pertaining to the conduct of this study must be retained by the investigator in the study site archive for at least 5 years after the end of the study unless local regulations or institutional policies require a longer retention period. No records may be destroyed during the retention period without the written approval of the sponsor. No records may be transferred to another location or party without written notification to the sponsor.

Investigator Documentation Responsibilities

All individual, patient-specific study data will also be entered into a 21 CFR Part 11-compliant electronic data capture (EDC) system on an eCRF in a timely fashion.

All data generated from external sources (e.g., laboratory and pharmacodynamic data), and transmitted to the sponsor or designee electronically, will be integrated with the patient's eCRF data in accordance with the data management plan.

An eCRF must be completed for each enrolled patient who undergoes any screening procedures, according to the eCRF completion instructions. The sponsor or CRO will review the supporting source documentation against the data entered into the eCRFs to verify the accuracy of the electronic data. The investigator will ensure that corrections are made to the eCRFs and that data queries are resolved in a timely fashion by the study staff.

The investigator will sign and date the eCRF via the EDC system's electronic signature procedure. These signatures will indicate that the investigator reviewed and approved the data on the eCRF, data queries, and site notifications.

Publications

The sponsor will comply with the requirements for publication of study results. In accordance with standard editorial and ethical practice, the sponsor will generally support publication of multicentre studies only in their entirety and not as individual site data.

Authorship will be determined by mutual agreement and in line with International Committee of Medical Journal Editors authorship requirements.

Appendix 6: Schedule of Assessments

Schedule of Assessments

		Screening	Baseline					EOT/ET			EOS
	Days	-21 to -1	1	8±1	15±1	22±1	29±1	36±1	57±7[a]	113±7[a]	169±7[a]
Assessment	Weeks			1	2	3	4	5	8	16	24
Informed consent		X									
Inclusion/exclusion criteria		X									
Demographics		X									
Body weight, height, and BMI		X									
Medical and surgical history		X									
Tumour sample[b]		X									
Serology[c]		X									
Pregnancy test[d]		X	X			X		X	X	X	X
**Safety**											
Concomitant medication		X	X	X	X	X	X	X	X	X	X
Adverse events/Serious adverse events		X	X	X	X	X	X	X	X	X	X
Clinical laboratory assessments[e]		X	X	X	X	X	X	X			
Vital signs[f]		X	X	X	X	X	X	X			
12-lead ECG		X	X	X	X	X	X	X			
Physical examination		X	X			X		X			
Injection site examination			X	X	X	X	X	X			
ECOG performance score		X	X			X		X			
**Pharmacodynamics**											
Survivin expression		X									
**Efficacy**											
Immunology sampling			X[g]	X[h]		X[h]		X	X	X	X
CT/MRI tumour assessments[i]		X							X	X	X
Bone scan[j]		X							X	X	X
CA-125[k]		X	X[g]						X	X	X
PSA and ALP[l]			X[g]						X	X	X

		Screening	Baseline					EOT/ET			EOS
	**Days**	**-21 to -1**	**1**	**8±1**	**15±1**	**22±1**	**29±1**	**36±1**	**57±7[a]**	**113±7[a]**	**169±7[a]**
**Assessment**	**Weeks**			**1**	**2**	**3**	**4**	**5**	**8**	**16**	**24**
**Other**											
OVM-200vaccination			X		X		X				
Survival									X	X	X

Abbreviations: ALP = total alkaline phosphatase; BMI = body mass index; CA-125 = cancer antigen-125; CT = computed tomography; ECG = electrocardiogram; ECOG = Eastern Cooperative Oncology Group; EOS = end of study; EOT = end of treatment; ET = early terminations; MRI = magnetic resonance imaging; NSCLC= non-small cell lung cancer; PSA = prostate-specific antigen.

[a] The window for the tumour assessment and bone scan is ±1 week.

[b] A suitable archival sample should be provided.

[c] Hepatitis B surface antigen and hepatitis C virus (HCV) ribonucleic acid or HCV antibodies. Human immunodeficiency virus antibodies only if there is a history.

[d] A urine pregnancy test for women of childbearing potential.

[e] Clinical chemistry, haematology, and urinalysis.

[f] Vital signs (heart rate, blood pressure, body temperature, and respiratory rate) will be obtained in the sitting position after the patient has rested for 5 minutes.

[g] Predose.

[h] In Phase 1a only.

[i] Tumour assessment will be performed using CT or MRI per Response Evaluation Criteria in Solid Tumour (RECIST) v1.1. The CT/MRI will include chest and abdomen for NSCLC patients; chest (in those patients with disease in the chest or upper abdomen lymphadenopathy at baseline), abdomen, and pelvis for ovarian cancer patients; and chest, abdomen, and pelvis for prostate cancer patients. Any patient with known or suspected brain metastases should have CT/MRI of the brain. Imaging will also include radionuclide bone scan for prostate cancer patients. Any NSCLC or ovarian cancer patients with bone lesions may have a bone scan during screening and repeat bone scans performed as clinically indicated. Postdose tumour assessments have a window of ±1 week.

[j] Radionuclide bone scan for prostate cancer patients. Any NSCLC or ovarian cancer patients with bone lesions may have a bone scan during screening and repeat bone scans performed as clinically indicated. Postdose bone scans have a window of ±1 week.

[k] Ovarian cancer patients only. Measured by the local lab.

[l] Prostate cancer patients only.
