# Supplementary material for: Survivin recombinant overlapping peptide (ROP) vaccine in advanced solid tumours: a first-in-human, multicentre, open-label, phase 1a dose-escalation study
Source: eClinicalMedicine. 2025 Dec 27;91:103717. doi: 10.1016/j.eclinm.2025.103717 (PMC12796589; doi:10.1016/j.eclinm.2025.103717)
Supplement: Statistical Analysis Plan [file mmc2.pdf]

Statistical Analysis Plan for Immunology Testing of Patient Samples in OVM-200-100

October 2025

Study Title

A Phase 1, Multicentre, Open-label, Nonrandomised, First-in-human Study of OVM-200 as a Therapeutic Vaccine in Patients with Locally Advanced or Metastatic Non-Small Cell Lung Cancer, Ovarian Cancer, and Prostate Cancer

Statistical Analysis Plan: **subsidiary part re immunology testing**
Reference: Statistical Analysis Plan 3.0 Fortrea
Statistical Analysis Plan Date: 30-June-2025
Investigational Product: OVM-200
Protocol Reference: OVM-200-100
Fortrea Study ID: 000000196384
Sponsor: Oxford Vacmedix UK Ltd.

Table of Contents-TBE

Glossary of Abbreviations

Abbreviation	Term
AE	Adverse Event
ALT	Alanine Aminotransferase
ALP	Alkaline Phosphatase
AST	Aspartate Aminotransferase
ATC	Anatomical Therapeutic Chemical
CA-125	Cancer Antigen 125
CI	Confidence Interval
CR	Complete Response
CT	Computed tomography
COVID-19	Coronavirus Disease of 2019
DBP	Diastolic Blood Pressure
DLT	Dose Limiting Toxicity
DoR	Duration of Response
ECG	Electrocardiogram
ECOG	Eastern Cooperative Oncology Group
eCRF	Electronic Case Report Form
ELISA	Enzyme-linked Immunosorbent Assay
ELISpot	Enzyme-linked Immune Absorbent Spot
EOS	End of Study
EOT	End of Treatment
GCIC	Gynaecological Cancer Intergroup
GGT	Gamma-Glutamyl Transferase
HR	Heart Rate
IPD	Important Protocol Deviations
MedDRA	Medical Dictionary for Regulatory Activities

Abbreviation	Term
MRI	Magnetic Resonance Imaging
MTD	Maximum Tolerated Dose
NCI-CTCAE	National Cancer Institute-Common Terminology Criteria for AEs
NSCLC	Non-small Cell Lung Cancer
ORR	Objective Response Rate
OS	Overall Survival
PCI	Potentially Clinically Important
PD	Progressive Disease
PFS	Progression Free Survival
PR	Partial Response
PSA	Prostate-Specific Antigen
PT	Preferred Term
QTcF	Fridericia corrected QT interval
RECIST	Response Evaluation Criteria in Solid Tumour
SAE	Serious AE
SAP	Statistical Analysis Plan
SBP	Systolic Blood Pressure
SD	Standard Deviation
SI	International System of Units
SMQ	Standardized MedDRA Query
SOC	System Organ Class
SRC	Safety Review Committee
TBD	To Be Determined
TFLs	Tables, Figures and Listings
TEAE	Treatment Emergent AEs
TTR	Time to Response
WHO	World Health Organization

Source documents

Document	Version	Date
Protocol	4.0	19-June-2023
eCRF	3.0	08-Feb-2024

Introduction

The full study is undertaken to investigate the use of a therapeutic vaccine in a group of patients with one of three cancer types known to overexpress survivin. This is in part confirmatory (safety at the proposed dosing) and exploratory (clinical/efficacy) as this is the first time this technology has been used in man. This document describes the analytical activities to be undertaken by the sponsor and should be read in conjunction with the main Fortrea SAP 3.0 referenced above.

Objectives of the Clinical Trial

The **primary** objective of the study is:

- To characterise the safety and tolerability of OVM-200 in patients with tumour types known to over-express survivin (non-small cell lung cancer [NSCLC], ovarian cancer, and prostate cancer).

The **secondary** objectives of the study are:

- To determine the immune response following administration of OVM-200 in patients with tumour types known to over-express survivin.
- To determine the recommended dose of OVM-200 for further development in patients with tumour types known to over-express survivin.

The **exploratory** objectives of the study are:

- To conduct a preliminary exploration of the antitumour activity of OVM-200 in patients with tumour types known to over-express survivin.
- To evaluate the correlation of survivin expression with response to the administration of OVM-200.

Study Design

This is a 2-part study in patients with:

i. NSCLC
ii. Ovarian cancer,
iii. Prostate cancer.

The first part (Phase 1a) comprises a first-in-human (FIH) multiple-dose, sequential-cohort 3+3 design to establish a dose of OVM-200 that is safe and tolerable, and that elicits an immune response in humans.

This dose will be taken forward into the second part (Phase 1b) of the study. Phase 1b will further assess the safety and tolerability of the selected dose and investigate the immune and tumour response in 3 expansion cohorts of additional patients with NSCLC, ovarian cancer, and prostate cancer respectively.

Phase 1a

OVM-200 will be administered after at least 1 line of systemic cancer treatment is completed, with the exception of supportive therapies and androgen deprivation therapies for prostate cancer patients, which may be continued throughout the study.

After a screening period of up to 21 days, eligible patients will arrive at the site on the first day of dosing (Day 1) to confirm eligibility, complete predose baseline assessments, and receive the first dose of OVM-200. Patients will receive 3 doses of OVM-200 at 2-week intervals. The patients will remain at the site for at least 4 hours after each dose for observation. Between dose administrations, patients will return for weekly visits through the end-of-treatment (EOT) visit on Week 5. There will be additional visits on Weeks 8 and 16 and an end-of-study (EOS) visit on Week 24.

A safety review committee (SRC) will review all available cumulative safety and immune response data throughout the trial.

Phase 1a will be a standard 3+3 design with up to 4 cohorts.

Three patients will initially be enrolled into a given dose cohort. A single sentinel patient will be dosed initially with 2 additional patients dosed at the same dose level at least 2 weeks later, if no dose-limiting toxicities (DLTs) or other safety and tolerability concerns are observed in the sentinel patient. If a DLT or other notable safety event is experienced in the sentinel patient, the SRC will review all available data before deciding on the continuation of dosing in subsequent patients.

If no DLT is observed in any of these patients to week 5 (1 week after the final vaccination in the last patient to be dosed) dosing will commence in the next cohort. If one patient develops a DLT at a specific dose, an additional three patients will be enrolled into that same dose cohort. Development of DLTs in more than 1 of 6 patients in a specific dose cohort suggests that the maximum tolerated dose (MTD) has been exceeded, and further dose escalation will not be pursued.

In addition, cohort 4 will not proceed if a clear plateau in immune response is observed in the previous 3 cohorts.

The SRC will review data from Phase 1a and make a recommendation on the Phase 1b dose based on safety and immune response. The dose in Phase 1b will not exceed the dose safely administered in Phase 1a.

Phase 1b

OVM-200 will be administered after at least 1 line of systemic cancer treatment is completed, with the exception of supportive therapies and androgen deprivation therapies for prostate cancer patients, which may be continued throughout the study.

After a screening period of up to 21 days, eligible patients will arrive at the site on the first day of dosing (Day 1) to confirm eligibility, complete predose baseline assessments, and receive the first dose of OVM-200. Patients will receive 3 doses of OVM-200 at 2-week intervals. Patients will remain at the site for at least 2 hours, if supported by the data from Phase 1a, after each dose for observation. Between dose administrations, patients will return for weekly visits through the EOT visit on Week 5.

Patients which in the opinion of the investigator could benefit from further treatment may continue with up to 8 additional immunisations (two weekly intervals) until they show disease progression or are considered not to be gaining further benefit in the opinion of the investigator. A modified schedule of events for additional visits is available in appendix 3.

Each immunisation visit has up to 3 components. Component 1 is the administration of the vaccine visit and surrounding procedures, component 2 is a 2-4 hours post-dose assessment (same day), and component 3 is a follow-up visit, 1 week after the first 3 immunisations, which includes immunology sampling (Component 3 is not applicable for 4th immunizations and beyond to patients who continue OVM-200 after the third injection, but will visit after 35 days)

After the last immunisation related visit is completed (either maximum number of administrations given or investigator/patient decision to withdraw from treatment), patients are followed up for up to 6 months after first immunisation.

Variables
The following patient criteria apply across the patient pool:

Age, gender, cancer diagnosis, stage at baseline, tumour cell surface expression of survivin, OR RECIST (Target and Non-target lesions).

Sampling variables across treatment and timing:

Dose (Ph1a only), days from first immunisation(D=0), number of immunisations. Samples will be collected for immunology testing as described below and will be tested for anti survivin IgG antibody titre (ELISA) and T cell activity by IGRA

Estimands

The number and percentage of patients with an immune response (as determined by ELISpot for T-cell responses and ELISA for antibody responses) by dose, therapeutic indication and overall in all patients in either phase who received OVM-200, regardless of the duration of treatment received and had at least 1 predose and 1 post-baseline sample taken to assess immune response.

Immune response is to be measured by enzyme-linked immune absorbent spot (IGRA/ELISpot) for T-cell responses and enzyme-linked immunosorbent assay (ELISA) for antibody responses. Immunological sampling will occur at baseline (pre-dose), Days 8 and 22 (phase 1a only) and at Days 36 (end of treatment; EOT), 57, 113 and 169 (end of study, EOS). Immune response will be calculated per dose level in phase 1a and per therapeutic indication in phase 1b. An immune response will be considered if, when using geometric means across 3 patients at each dose level/therapeutic indication, a difference between pre-immunisation and post immunisation is calculated to be 2 SDs or more. Missing immune response assessments will not be imputed.

Numbers of patients

Phase 1a: 3 to 24 patients will be studied in 1 to 4 dose cohorts of 3-6 patients each.

Phase 1b: A minimum of 24 patients will be studied in 3 therapeutic indication cohorts of approximately 8 patients each. More than 8 patients may be enrolled in a cohort, for a maximum of 28 patients total in this phase of the study. If more than 8 patients are enrolled in 1 of the cohorts (up to a maximum of 10 patients), fewer patients may be enrolled in either or both of the other cohorts.

All patients are sampled and tested in this study

Sample size
Phase 1a: No formal sample size calculation has been performed. The sample size is based primarily upon clinical considerations and is typical for first-in-human, oncologic, dose-escalation studies.
Phase 1b: The sample size is based primarily upon clinical considerations. However, in terms of the overall assessment of immune response and RECIST response, 24 patients overall will allow for evaluation of response rates with an approximate precision of 10% in the worst-case scenario.

**Patient**	**Number**	**D**	**Dose**	**PSA**	**ALP**	**CA-125**	**Recist**	**Ab Titre**	**T-cell score**	**Safety**	Age	# Immunisatios	Gender	BMI	IHC Status	Circulating S	Cancer	Stage	Prior treatment
		Screening																	
		D1																	
		D8																	
		D15																	
		D29																	
		D35																	
		D43																	
		D57																	
		D71																	
		D85																	
		D99																	
		D113																	
		D169																	

These will allow analysis of immunogenic development and investigation of any emerging correlations.

In principle and where possible immune response will be calculated by phase, dose/therapeutic indication, and overall with 80 and 90% exact confidence intervals (CIs) using the Immune Response Evaluable Population. The number and percentage of patients with immune response will be summarised by phase, dose/therapeutic indication, and overall and timepoint.

In accordance with the baseline value definition in Section 5.1.2, the absolute and percentage change from baseline will be derived as follows:

Absolute change (unit) = (post-baseline value – baseline value)
Percentage change from baseline = [(post-baseline value– baseline value) / baseline value] × 100

Immune response will be listed by phase, dose/therapeutic indication and timepoint using the Immune Response Evaluable Population and will included the absolute and percentage change from baseline.

Appendix 1

Source Documents
The SAP was written based on and in association with the following documentation from Fortrea:
Document Version Date
Protocol 4.0 19-June-2023
eCRF 3.0 08-Feb-2024
SAP3.0 (fortrea)

Appendix 2

Exploratory Efficacy Variable(s) (not covered by this plan)

Tumour assessment will be performed using computed tomography (CT) or magnetic resonance imaging (MRI) at screening (day -21 to -1), day 57, day 113 and day 169. The following exploratory variables will be defined using tumour assessment per RECIST v1.1:

• Objective response rate (ORR) is defined as the proportion of patients who achieved CR and/or PR per local investigator assessment using RECIST.
• Time to response (TTR) is defined for patients with CR or PR as the time between the first dose of OVM-200 and the first observation of tumour response (CR or PR) per local investigator assessment using RECIST. Patients who do not experience CR or PR will be censored at the time of their last RECIST assessment according to Table 2 in Section 6.6.3.2.
• Duration of response (DoR) is defined as the time between the first observation of tumour response (CR or PR) and tumour progression (bone or soft tissue) or death, whichever comes first, using local investigator assessment using RECIST (i.e. date of tumour progression, death or censoring – date of response + 1). Patients who do not experience disease progression or death will be censored at the time of their last RECIST assessment. Patients who start new anticancer treatment will be censored at the time of their last RECIST assessment before starting new anticancer treatment.
• Progression free survival (PFS) is defined as the time from study entry until tumour progression (bone or soft tissue) or death, whichever comes first, using local investigator assessment using RECIST (i.e. date of tumour progression, death or censoring – date of first dose of OVM-200 + 1). Patients who do not experience disease progression or death will be censored at the time of their last RECIST assessment. Patients who start new anticancer treatment will be censored at the time of their last RECIST assessment before starting new anticancer treatment. In addition, a sensitivity analysis will be conducted where patients who start a new anticancer treatment will be considered to have progressed at the date the new anticancer treatment was started.
• Overall survival (OS) is defined as the time from first dose of OVM-200 until death due to any cause (i.e. date of death or censoring – date of first dose of OVM-200 + 1). Patients who are alive will be censored at the time of their last site contact. For patients who complete the study, this will be administrative censoring at the date they are last known to be alive.

In prostate cancer patients, the following exploratory efficacy variables will be defined:
• Prostate-specific antigen (PSA) response is defined as a decrease of ≥ 50% from baseline values. Patients who do not have evaluable PSA data will be treated as non-response.
• Total Alkaline Phosphatase (ALP) response is defined as a decrease of > 30% from baseline values. Patients who do not have evaluable ALP response data will be treated as non-response.
In ovarian cancer patients:
• Cancer antigen 125 (CA-125) response will be defined according to the Gynaecological Cancer Intergroup (GCIC) criteria. Patients will be evaluable if a pre-dose CA-125 level (taken with 2 weeks prior to starting study drug) is ≥ 2 × ULN, if there is no more than a 10% reduction in CA-125 between the two predose measurements and if the same assay method is used for each sample from each patient. A response will be considered to have occurred if there is ≥ 50% reduction in CA-125 levels from the last predose sample. If response is not evaluable then patients will be treated as non-response.

Appendix 3

Time Points and Visit Windows

General Definitions

All assessment days will be related to the first day of first dose of OVM-200.
Day 1 is defined as first dose of OVM-200. Relative days after Day 1 are calculated as (assessment date – Day 1 date) + 1. Relative days prior to Day 1 are calculated as (assessment date – Day 1 date). The day prior to Day 1 is Day -1. Day 0 is not defined.
The date of the first dose of OVM-200 for each patient will be taken from the Study Drug Administration electronic case report form (eCRF) page.
The date of the last dose of OVM-200 for each patient will be calculated from the Study Drug Administration eCRF page as the latest date per patient.

Screening Period

For all patients, the screening period is defined as the period from informed consent to the first dose of OVM-200. For some variables, data from more than one assessment within the screening period can be collected prior to the first dose of OVM-200. Therefore, the baseline value for a variable is defined as the last non-missing value collected in the screening period before the first dose of OVM-200.

Treatment Period

Data collected at Day 1 will be assigned to the Treatment Period unless the time (HH:MM) of data collection and time (HH:MM) of first dose of OVM-200 are both recorded and the data collection time is before the time of first dose of OVM-200. In this case, the assessment will be assigned to the screening period. If the time (HH:MM) of data collection is not recorded but the protocol and / or eCRF includes an instruction to the effect that all Day 1 assessments are to be performed prior to the first dose of OVM-200, the data collected at Day 1 will be assigned to the screening period. However, adverse events and medications starting on Day 1, will be assigned to the Treatment Period. The Treatment Period is defined as the period from the date / time of the first dose of OVM-200 up to and including the end of treatment visit date.

Appendix 4

Schedule of treatment

	**Screening**	**Baseline**	**2-4 hours post-dose**			**2-4 hours post-dose**			**2-4 hours post-dose**	**EOT/ ET**			**EOS**

Assessment / Days	-21 to -1	1	1	8±1	15±1	15±1	22±1	29±1	29±1	36±1	57±7[a]	113±7[a]	169±7[a]
Weeks				1	2	2	3	4	4	5	8	16	24
Informed consent	X												
Inclusion/exclusion criteria	X												
Demographics	X												
Body weight, height, and BMI	X												
Medical and surgical history	X												
Tumour sample[b]	X												
Serology[c]	X												
Pregnancy test[d]	X	X					X			X	X	X	X
**Safety**													
Concomitant medication	X	X	X	X	X	X	X	X	X	X	X	X	X
Adverse events/Serious adverse events	X	X	X	X	X	X	X	X	X	X	X	X[o]	X[o]
Clinical laboratory assessments[e]	X	X		X	X		X	X		X			
Vital signs[f]	X	X	X	X	X	X	X	X	X	X			
12-lead ECG[i]	X	X	X	X	X	X	X	X	X	X			
Physical examination	X	X					X			X			
Injection site examination[j]		X	X	X	X	X	X	X	X	X			
ECOG performance score	X	X					X			X			
**Pharmacodynamics**													
Survivin expression	X												
**Efficacy**													
Immunology sampling		X[g]		X[h]			X[h]			X	X	X	X
CT/MRI tumour assessments[k]	X										X	X	X
	**Screening**	**Baseline**	**2-4 hours post-dose**			**2-4 hours post-dose**			**2-4 hours post-dose**	**EOT/ET**			**EOS**

	Days	-21 to -1	1	1	8±1	15 ±1	15±1	22 ±1	29 ±1	29±1	36±1	57±7[a]	113±7[a]	169±7[a]
**Assessment**	**Weeks**				**1**	**2**	**2**	**3**	**4**	**4**	**5**	**8**	**16**	**24**
Bone scan[l]		X										X	X	X
CA-125[m]		X	X[g]									X	X	X
PSA and ALP[n]			X[g]									X	X	X
**Other**														
OVM-200 vaccination			X			X			X					
Survival												X	X	X

			**2-4 hours post-dose**			**EOS**
**Assessment**	**Days (Weeks)**	**43 ±1 (6)**<br>**57 ±1 (8)**<br>**71 ±1 (10)**<br>**85 ±1 (12)**<br>**99 ±1 (14)**<br>**113 ±1 (16)**<br>**127 ±1 (18)**<br>**141 ±1 (20)**	**43 ±1 (6)**<br>**57 ±1 (8)**<br>**71 ±1 (10)**<br>**85 ±1 (12)**<br>**99 ±1 (14)**<br>**113 ±1 (16)**<br>**127 ±1 (18)**<br>**141 ±1 (20)**	**57 ±7 (Week 8)** (as per Appendix 6, Week 8)	**113 ±7 (Week 16)** (as per Appendix 6, Week 16)	**169 ±7 (Week 24)** (as per Appendix 6, Week 24)
**Safety**						
Pregnancy test[a]		X		X	X	X
Concomitant medication		X	X	X	X	X
Adverse events/Serious adverse events		X	X	X	X[j]	X[j]
Clinical laboratory assessments[b]		X				
Vital signs[c]		X	X			
12-lead ECG[d]		X	X			
Physical examination						
Injection site examination[e]		X	X			
ECOG performance score						
**Efficacy**						
Immunology sampling				X	X	X
CT/MRI tumour assessments[f]				X	X	X
Bone scan[g]				X	X	X
CA-125[h]				X	X	X
PSA and ALP[i]				X	X	X

			**2-4 hours post-dose**			**EOS**
**Assessment**	**Days (Weeks)**	**43 ±1 (6)**<br>**57 ±1 (8)**<br>**71 ±1 (10)**<br>**85 ±1 (12)**<br>**99 ±1 (14)**<br>**113 ±1 (16)**<br>**127 ±1 (18)**<br>**141 ±1 (20)**	**43 ±1 (6)**<br>**57 ±1 (8)**<br>**71 ±1 (10)**<br>**85 ±1 (12)**<br>**99 ±1 (14)**<br>**113 ±1 (16)**<br>**127 ±1 (18)**<br>**141 ±1 (20)**	**57 ±7 (Week 8)**<br>(as per Appendix 6, Week 8)	**113 ±7 (Week 16)**<br>(as per Appendix 6, Week 16)	**169 ±7 (Week 24)**<br>(as per Appendix 6, Week 24)
**Other**						
OVM-200 vaccination		X				
Survival				X	X	X
