# Supplementary material for: Survivin recombinant overlapping peptide (ROP) vaccine in advanced solid tumours: a first-in-human, multicentre, open-label, phase 1a dose-escalation study
Source: eClinicalMedicine. 2025 Dec 27;91:103717. doi: 10.1016/j.eclinm.2025.103717 (PMC12796589; doi:10.1016/j.eclinm.2025.103717)
Supplement: Supplementary Materials [file mmc3.docx]

**Supplementary Materials**

**Table S1**. The overlapping synthetic peptides pool used as a test stimulator.

| **Peptide reference no.** | **Batch Number** | **Amino acid sequence** | **MW (Da)** |
| --- | --- | --- | --- |
| CPD67190 | P210511-LL896229 | MGAPTLPPAWQPFLKDHRISTFKNWPFLEG | 3483 |
| CPD67191 | P210511-LL896231 | DHRISTFK.NWPFLEGCACTPERMAEAGFIH | 3464.9 |
| CPD67192 | P210511-LL896232 | ACTPERMAEAGFIHCPTENEPDLAQCFF | 3128.49 |
| CPD67193 | P210511-LL896233 | PTENEPDLAQCFFCFKELEGWEPDDDPIE | 3414.63 |
| CPD67194 | P210511-LL896234 | FKELEGWEPDDDPIEEHKKHSSGCAFLSVK | 3458.76 |
| CPD67195 | P210511-LL896235 | EHKKHSSGCAFLSVKKQFEELTLGEFLK | 3221.68 |
| CPD67196 | P210511-LL896236 | QFEELTLGEFLKLDRERAKNKIAKETNNK | 3463.89 |
| CPD67197 | P210511-LL896237 | RERAKNKIAKETNNKKKEFEETAEKVRRAI | 3616.09 |
| CPD67198 | P210511-LL896238 | KEFEETAEKVRRAIEQLAAMD | 2464.74 |

MW: molecular weight

**Table S2**. Median anti-survivin IgG titre (log-transformed) developed in each dose group (250, 500, 1000, and 2000 µg) at different time points.

| **Time points** | **Dose groups** | | | |
| --- | --- | --- | --- | --- |
|  | **250 µg**  **(n=3)** | **500 µg**  **(n=3)** | **1000 µg**  **(n=3)** | **2000 µg**  **(n=3)** |
| Day 1 | 0 | 0 | 5.32 | 4.32 |
| Day 8 | 0 | 0 | 5.32 | 5.32 |
| Day 22 | 4.32 | 6.32 | 7.32 | 6.32 |
| Day 36 (EOT) | 5.32 | 5.66 | 14.32 | 12.32 |
| Day 57 | 5.16 | 8.82 | 14.32 | 14.32 |
| Day 113 | 11.32 | 13.32 | 15.32 | 17.32 |
| Day 169 (EOS) | NA | 14.32 | NA | 16.32 |

**Table S3**. Median of ELISpot T cell response (SFU per million cells) developed in each dose group (250, 500, 1000, and 2000 µg) at different time points.

| **Time points** | **Dose groups** | | | |
| --- | --- | --- | --- | --- |
|  | **250 µg**  **(n=3)** | **500 µg**  **(n=3)** | **1000 µg**  **(n=3)** | **2000 µg**  **(n=3)** |
| Day 1 | 14 | 27 | 243 | 50 |
| Day 8 | 32 | 0 | 60 | 0 |
| Day 22 | 20 | 475 | 30 | 1282 |
| Day 36 (EOT) | NA | 39 | 83 | 678.5 |
| Day 57 | 47 | 33 | 22 | 80 |
| Day 113 | 23 | 313 | 0 | 189 |
| Day 169 (EOS) | NA | 38 | NA | 110 |
